# Deletion of the Wilms’ Tumor Suppressor Gene in the Cardiac Troponin-T Lineage Reveals Novel Functions of WT1 in Heart Development

**DOI:** 10.3389/fcell.2021.683861

**Published:** 2021-07-22

**Authors:** Sandra Díaz del Moral, Silvia Barrena, Francisco Hernández-Torres, Amelia Aránega, José Manuel Villaescusa, Juan José Gómez Doblas, Diego Franco, Manuel Jiménez-Navarro, Ramón Muñoz-Chápuli, Rita Carmona

**Affiliations:** ^1^Department of Animal Biology, University of Málaga, Málaga, Spain; ^2^Department of Biochemistry and Molecular Biology III and Immunology, Faculty of Medicine, University of Granada, Granada, Spain; ^3^Medina Foundation, Technology Park of Health Sciences, Granada, Spain; ^4^Department of Experimental Biology, Faculty of Experimental Sciences, University of Jaén, Jaén, Spain; ^5^Heart Area Clinical Management Unit, University Hosp tal Virgen de la Victoria, CIBERCV Enfermedades Cardiovasculares Health Institute Carlos III, Biomedical Research Institute of Malaga (IBIMA), University of Málaga, Málaga, Spain

**Keywords:** Wilms’ tumor suppressor gene, cardiomyocytes, cardiac development, calcium homeostasis, potassium channels

## Abstract

Expression of Wilms’ tumor suppressor transcription factor (WT1) in the embryonic epicardium is essential for cardiac development, but its myocardial expression is little known. We have found that WT1 is expressed at low levels in 20–25% of the embryonic cardiomyocytes. Conditional ablation of WT1 using a cardiac troponin T driver (Tnnt2^*Cre*^) caused abnormal sinus venosus and atrium development, lack of pectinate muscles, thin ventricular myocardium and, in some cases, interventricular septum and cardiac wall defects, ventricular diverticula and aneurisms. Coronary development was normal and there was not embryonic lethality, although survival of adult mutant mice was reduced probably due to perinatal mortality. Adult mutant mice showed electrocardiographic anomalies, including increased RR and QRS intervals, and decreased PR intervals. RNASeq analysis identified differential expression of 137 genes in the E13.5 mutant heart as compared to controls. GO functional enrichment analysis suggested that both calcium ion regulation and modulation of potassium channels are deeply altered in the mutant myocardium. In summary, together with its essential function in the embryonic epicardium, myocardial WT1 expression is also required for normal cardiac development.

## Introduction

The Wilms tumor suppressor gene (*Wt1*) encodes a C2H2-type zinc-finger transcription factor that appears in mammals under different isoforms, participating in transcriptional regulation, RNA metabolism and protein-protein interactions. WT1 is involved in the development of a number of organs, including kidneys and gonads, spleen, adrenals, liver and diaphragm (Hastie, 2017). Systemic loss of function of WT1 in mice causes embryonic lethality at midgestation, a lethality attributed to defects in cardiac development (Martínez-Estrada et al., 2010; Cano et al., 2016). WT1 is highly expressed in the embryonic epicardium where it regulates a process of epicardial-mesenchymal transformation and the development of the epicardial-derived cells that contribute with fibroblasts and smooth muscle to the cardiac connective and vascular tissues (Martínez-Estrada et al., 2010; Von Gise et al., 2011). Furthermore, the epicardium is a source of signals for myocardial proliferation and the development of the compact ventricular wall. The phenotype of WT1 loss of function in mice involves thinning of the myocardial walls associated to a defective coronary development, the most probable cause of the embryonic death (Lavine et al., 2005; Vega-Hernández et al., 2011).

The cardiac phenotype of WT1 loss of function had hitherto been exclusively related with its prominent epicardial expression. However, we have collected experimental evidence suggesting that WT1 is also expressed at lower levels in a fraction of the embryonic cardiomyocytes. This myocardial expression of WT1 is hitherto poorly known, and mentioned just in a few papers (Villa del Campo et al., 2016; Van Eif et al., 2020; Wagner et al., 2021). Rudat and Kispert (2012) did not exclude a weak myocardial expression of WT1 at E9.5 using the Wt1^TM 1(*EGFP/cre*)*Wtp*^ driver. Velecela et al. (2019) described a population of cells in the embryonic heart expressing low levels of the reporter GFP in a *Wt1*^*GFP*^ knockin mouse model. This population expressed a number of myocardial genes but the authors conclude that further research was needed in order to characterize the nature of this population of cells presumably expressing WT1. A better characterization of this presumptive myocardial expression of WT1 is also relevant since a lineage of cardiomyocytes derived from WT1-expressing cells was considered as arising from epicardial progenitors (Zhou et al., 2008), giving rise to a controversy about the myocardiogenic potential of epicardial-derived cells (Cai et al., 2008; Rudat and Kispert, 2012).

The aim of this report is to study in depth the myocardial expression of WT1 and to investigate if conditional deletion of WT1 in embryonic cardiomyocytes causes alterations in cardiac development, anomalies that could have been hitherto masked in previous descriptions of systemic or epicardial-specific loss of function.

## Materials and Methods

### Animal Models

The animals used in our research program were handled in compliance with the institutional and European Union guidelines (Directive 2010/63/EU of the European Parliament) for animal care and welfare. The procedures used in this study were approved by the Committee on Ethics of Animal Experiments of the University of Málaga (procedure code 2018-0018). According to these procedures, the mice were euthanized by cervical dislocation. For electrocardiography, animals were anesthetized with Ketamine, 64 μg/g body weight and Xylazine 10 μg/g in PBS (i.p.). For cardiac MRI, animals were anesthetized with a 1% isoflurane in medical air at a flow rate of 1 L/min in an induction chamber and then transferred to the MRI cradle. Respiratory rate was monitored throughout the MRI experiment and the anesthesia flow was readjusted when needed to maintain a rate of 60 ± 10 bpm.

The WT1^*GFP*^ knockin line (Hosen et al., 2007) in which the exon 1 of a *Wt1* allele has been replaced by the GFP sequence was used as a reporter for active WT1 transcription. The Tg(WT1-cre)#Jbeb mouse line (WT1^*Cre*^ from now on) has been used in previous studies to trace the WT1-expressing cell lineage or to delete specific genes in WT1-expressing cells (Del Monte et al., 2011; Wessels et al., 2012; Cano et al., 2013, 2016; Casanova et al., 2013). The generation of G2-GATA4^*Cre*^ line has been described elsewhere (Rojas et al., 2005). This driver allows conditional deletion of genes in the lateral mesoderm including proepicardium and epicardium (Cano et al., 2016). For cardiac troponin T (Tnnt2 from now on) lineage tracing and conditional deletion of WT1 in cardiomyocytes, we have used the Tg(Tnnt2-cre)5Blh mouse strain. The Tnnt2 promoter drives early expression of Cre recombinase in the cardiomyocyte lineage beginning at E7.5 (Jiao et al., 2003). We have also described the activation of this driver and its recombination in a fraction of the proepicardial cells (Carmona et al., 2020) as described below. Crossing this strain with the *Wt1*^*LoxP*^ mice [described in Martínez-Estrada et al. (2010)] causes WT1 deletion in the Tnnt2-expressing cell lineage. In the context of this paper, we will call “mutant” to all the mice with a Tnnt2^*Cre/+*^;W1^*flox/flox*^ genotype.

For lineage tracing studies, the murine lines carrying the WT1^*Cre*^ and the Tnnt2^*Cre*^ drivers were crossed with homozygote Rosa26EYFP [B6.129 × 1-Gt(ROSA)26^*Sortm*1(*EYFP*)*Cos/J*^] mice (Srinivas et al., 2001) to generate permanent reporter expression in the lineages of WT1 and Tnnt2-expressing cells.

Embryos were staged from the time point of vaginal plug observation, which was designated as the stage E0.5. Whole embryos were excised from uterus, washed in PBS and dissected to obtain the hearts or fixed in 4% fresh paraformaldehyde solution in PBS for 2–8 h. Fixed embryos were paraffin-embedded or washed in PBS. Adult hearts were fixed for 4–6 h following the same procedure.

### Analytical Flow Cytometry

For flow cytometry analysis, dissected hearts were dissociated for 20 min at 37°C in pre-warmed 0.1% collagenase (C9407, Sigma) solution in PBS and homogenized by repeated pipetting. Cell suspensions were washed in cytometry buffer (PBS plus 2% fetal bovine serum and 10 mM HEPES) and filtered through a 70 μm nylon mesh. Then, cells were incubated on ice in the dark with the fluorochrome-conjugated antibodies. DAPI staining showed that less than 10% of the cells were damaged.

Cardiomyocytes of E10.5–E15.5 embryos were identified by VCAM-1 immunostaining, since this cell adhesion molecule is only expressed by the myocardium in early developmental stages (Pontén et al., 2013, see also Supplementary Figure 1). Cardiomyocytes from E15.5–E17.5 embryos were identified using a mitochondrial staining reagent (Mitotracker Deep Red, Invitrogen, M22426) (Hattori et al., 2010). We incubated the cell suspension in the Mitotracker solution (100 nM in cytometry buffer) at 37°C for 30 min.

Cells were analyzed in a FACS Verse flow cytometer. Data were analyzed with Kaluza Analysis software (version 2.1) and displayed on tables as mean ± standard error of mean. Negative controls (Cre-negative littermates) and isotypic antibodies allowed setting of the gates.

Details of the antibodies used for flow cytometry are provided in Supplementary Table 1.

### Cell Culture

Hearts from E10.5 wildtype embryos were dissected and minced in sterile Ca^2+^ and Mg^2+^-free PBS with 20 mM of 2,3-butanedione 2-monoxime (2,3-BDM; Sigma #B0753). Then, the fragments were digested in Medium 199 with 20 mM of BDM and 0.2 mg/mL trypsin for 5 min at 37°C. The cell suspension was washed with high glucose DMEM:M199 (0.7:0.3), plus 10% horse serum, 0.5% FBS and penicillin/streptomycin. The cells were cultured with 5% CO_2_ for 48 h, and were then fixed in 4% paraformaldehyde for 10 min and processed for immunofluorescence.

### Immunofluorescence and Confocal Microscopy

Deparaffinized sections or fixed cultured cells were washed in Tris-PBS (TPBS) and blocked for non-specific binding with SBT (16% sheep serum, 1% bovine albumin, and 0.1% Triton X-100 in TPBS). When biotinylated secondary antibodies were used, endogenous biotin was blocked with the Avidin-Biotin blocking kit from Vector. Single immunofluorescence was performed incubating the sections with the primary antibody overnight at 4°C, washing in TPBS and incubating with the corresponding fluorochrome-conjugated secondary antibody for 1 h at room temperature. Double immunofluorescence was performed by mixing both primary antibodies (rabbit polyclonal and mouse/rat monoclonal), and incubating overnight at 4°C. We then used a biotin-conjugated secondary antibody, followed by 45 min incubation with Cy5-conjugated streptavidin. Nuclei were counterstained with DAPI (Sigma, D9542). Sections were mounted in PBS:Glycerol 1:1.

Details of the antibodies used for immunofluorescence are provided in Supplementary Table 1.

### Analysis of Trabeculation and Proliferation

Histological sections of E13.5 mutant and control hearts were used to assess the rate of proliferation and the relative thickness of the compact and the trabeculated ventricular layers. For cell proliferation, pregnant females were intraperitoneally injected with 3 mg of BrdU (B5002, Sigma) diluted in 300 μL of PBS. Two hours after the injection the mice were euthanized and the embryos were processed for BrdU immunofluorescence as described above. Confocal images were analyzed by counting the number of BrdU+ cells in the section and measuring the total area of the nuclei stained with DAPI (1/1000) using ImageJ software. The mean area of the nuclei was estimated, and the total number of nuclei in the section was estimated dividing the total DAPI stained area by the mean area of the nuclei. Percentages of BrdU+ nuclei were calculated for each tissue section. We analyzed 29 sections of six wildtype and 15 sections of three mutant embryos.

The degree of ventricular compaction was estimated by dividing the thickness of the compact layer by the total thickness of the cardiac wall (compact plus trabeculated), in the midpoint between the base and the apex of the ventricle. We analyzed 40 histological sections from 12 control and 18 mutant E13.5 embryos, and 5 control and 5 mutant E15.5 embryos.

### ECG and MRI

Electrocardiograms were obtained from anesthetized mice using the data acquisition system BioAmp PowerLab 8/35 (ADInstruments). Negative and positive electrodes were placed in the upper right and lower left limbs, respectively, and the neutral pole was placed in the lower right limb. We analyzed a section of the ECG (about 1 min) using the ECG module of the LabChart 8.0 software and the preset mouse settings.

MRI experiments were carried on a Bruker BioSpec MRI system (Bruker BioSpec, Bruker BioSpin, Ettlingen, Germany) equipped with 400 mT m-1 field gradients and a 40 mm quadrature bird-cage resonator. Cine short-axis (SAX) and long-axis (LAX) were acquired using a retrospective self-gated FLASH sequences (IntraGate, Bruker BioSpin MRI, Ettlingen, Germany). The acquisition parameters were as follows: α = 20°, TE = 2.88 ms, TR = 8.5 ms, FOV = 11 × 22 mm, matrix size = 192 × 384, movie frames = 16, number of slices = 1, and slice thickness = 0.8.

### qPCR

Total RNA was extracted from whole hearts of Tnnt2^*Cre+/–*^; Wt1^*flox/flox*^ (mutant) and Tnnt2^*Cre–/–*^; Wt1^*flox/flox*^ (control) E13.5 embryos using NucleoSpin RNA XS (Macherey Nagel) according to the manufacturer’s instructions and quantified by a Nanodrop ND-1000 Spectrophotometer. Real time PCR experiments were performed with 20 ng of cDNA, SsoFast EvaGreen-mix (Bio-Rad, Hercules. CA, United States) and corresponding primer sets. qPCRs were performed using a LightCycler^®^ 96 thermocycler (Roche). Gapdh and β-actin were used as internal controls. Analysis of relative gene expression data was performed using the 2^–ΔΔ*C*^*^*T*^* method (Livak and Schmittgen, 2001). Each PCR reaction was carried out in triplicate and repeated in at least three distinct biological samples (a whole E13.5 heart for sample) to obtain representative means. Sequences of the primers used for qPCR are provided in Supplementary Table 2.

### RNASeq

RNA-Seq analysis was performed on three wildtype (Tnnt2^*Cre–/–*^; Wt1^*flox/flox*)^ and three mutant E13.5 embryos. Total RNA from each heart was extracted by using RNAqueous^TM^-Micro Total RNA Isolation Kit (Ambion, AM1931) following manufacturer’s instructions. Transcriptome analysis of total RNA was performed by Novogene^1^. Only RNA samples with RNA integrity number (RIN) higher than 8.5 were selected for libraries preparation. 200 ng of total RNA was subsequently used to prepare RNA-Seq library by using TruSeq RNA sample prep kit (Illumina) according to manufacturer’s instructions. Paired-end (2 × 150) RNA sequencing was performed on Illumina NovaSeq 6000 platform (Illumina).

For trimming and alignment of raw data, fastq sequences reads were uploaded to the European version of Galaxy platform (Afgan et al., 2016). Reads were trimmed with Trim Galore software (Galaxy Version 0.4.3.1) and aligned to the built-in mouse reference genome mm10 with RNA STAR Gapped-read mapper for RNA-seq data (Galaxy Version 2.6.0b-2) (Dobin et al., 2013).

For gene expression analysis, bam files were downloaded from Galaxy server and analyzed with different RStudio version 1.1463, R version 3.4.4 packages downloaded from Bioconductor website^2^. Reads were assigned to genes by using “featureCounts” function from package “Rsubread” (Liao et al., 2019) version 1.28.1 and mouse annotation file release M19, GRCm38.p6. Uniquely, mapped reads were used to calculate gene expression. Differential gene expression analysis was performed through package “DESeq2” version 1.26.0 (Love et al., 2014). All gene comparison with padj <0.05 (Benjamini & Hochberg test) were considered differentially expressed among experimental conditions. GO Functional Enrichment Analysis was performed through Over Representation Analysis (ORA) Method (Boyle et al., 2004) by using “clusterProfiler” v.3.6.0. package (Yu et al., 2012). Gene sets with *p*-value < 0.05 were considered over-represented among experimental conditions.

### Statistics

Statistical comparison of means was performed using two-tailed Student’s *t*–test when the data were normally distributed (Kolmogorov-Smirnov test). Mann-Whitney *U*-test was used when data differed significantly from a normal distribution. A Chi-square test was used for comparison between observed and expected frequencies of mutants. We considered *p* < 0.05 as the criterion for statistical significance.

## Results

### WT1 Is Expressed in a Fraction of Cardiomyocytes During Development

**FIGURE 1:**
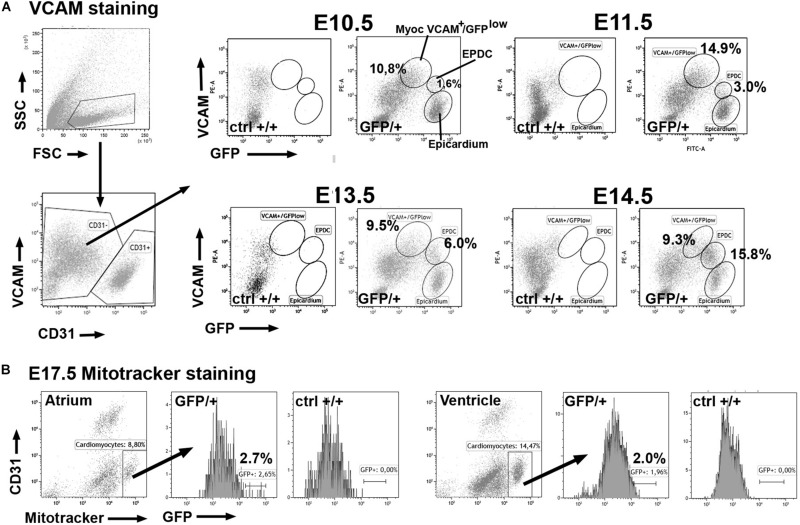
Expression of the GFP reporter in cardiomyocytes of the WT1^*GFP/+*^ knockin line (GFP/ + = heterozygous embryos; ctrl + / + = homozygous negative controls). Representative cytograms obtained from the analysis of 29 embryos. **(A)** Cardiomyocytes can be identified as VCAM+ /CD31- cells in the earliest stages of cardiac development. A fraction of these cardiomyocytes expresses the GFP reporter (top row) at lower levels than epicardial cells. In stages E13.5–E15.5 the reporter expression decreases in the VCAM + population. Note the increasing population of VCAM + expressing cells that represent the epicardial-derived mesenchymal cells (EPDC). **(B)** Stage E17.5. Cardiomyocytes can be identified by Mitotracker staining in this late developmental stage. A small fraction of atrial and ventricular cardiomyocytes still expresses the GFP reporter.

The *Wt1*^*GFP*^ knockin mouse model (heterozygote, GFP/ + embryos) allowed us to report activation of the *Wt1* locus. Using analytical flow cytometry we have found that a fraction of the non-endothelial cells of the developing heart shows some degree of GFP expression (Figure 1). Epicardial cells can be easily recognized by their strong GFP expression (Velecela et al., 2019). Myocardial cells of the early developmental stages can be safely identified by VCAM immunoreactivity, absent in endocardial and epicardial cells (Pontén et al., 2013, see also Supplementary Figure 1). A fraction of the VCAM+ cardiomyocytes (about 13% of the non-endothelial cells) express low levels of GFP at E10.5 (Table 1). This percentage decreases at E14.5–E15.5 (Figure 1A and Table 1). As the myocardium reduces its GFP expression, a population of epicardial-derived mesenchymal cells (EPDC) appears and progressively acquires VCAM immunoreactivity (Figure 1A and Supplementary Figure 2). Due to this emerging VCAM expression in non-myocardial cells, we identified cardiomyocytes at late stages by their mitochondrial content (Mitotracker staining, Hattori et al., 2010) and we confirmed the decrease in the myocardial expression of WT1. Only a small fraction of Mitotracker-positive cardiomyocytes (2% in the ventricles, 4% in the atria) showed activation of the WT1 reporter at E17.5 (Figure 1B and Table 1).

**TABLE 1:** Percentage of cardiomyocytes (defined as indicated in the phenotype column) expressing the reporter GFP (knockin line *Wt1*^*GFP/+*^) or YFP (lineage tracing line Wt1^*Cre*^; R26R^*EYFP*^) (mean ± s.e.m.).

Model	Stage	Phenotype	% Cardiomyocytes	N
WT1^*GFP*^	E10.5	CD31-/VCAM + /GFP^*low*^	13.5 ± 1.2	4
	E11.5	CD31-/VCAM + /GFP^*low*^	13.7 ± 1.6	4
	E13.5	CD31-/VCAM + /GFP^*low*^	10.8 ± 0.7	4
	E14.5	CD31-/VCAM + /GFP^*low*^	7.6 ± 0.6	5
	E15.5	CD31-/VCAM + /GFP^*low*^	3.5 ± 0.6	4
	E17.5 Atria	CD31-/Mitotracker high/GFP^*low*^	4.2 ± 0.6	4
	E17.5 Ventricles	CD31-/Mitotracker high/GFP^*low*^	2.2 ± 0.4	4
WT1^*Cre*^;R26R^*EYFP*^	E12.5	CD31-/VCAM + /YFP +	17.7 ± 5.1	4
	E15.5 Atria	CD31-/Mitotracker^*high*^/YFP +	23.6 ± 7.0	3
	E15.5 Ventricles	CD31-/Mitotracker^*high*^/YFP +	26.4 ± 4.4	4

**FIGURE 2:**
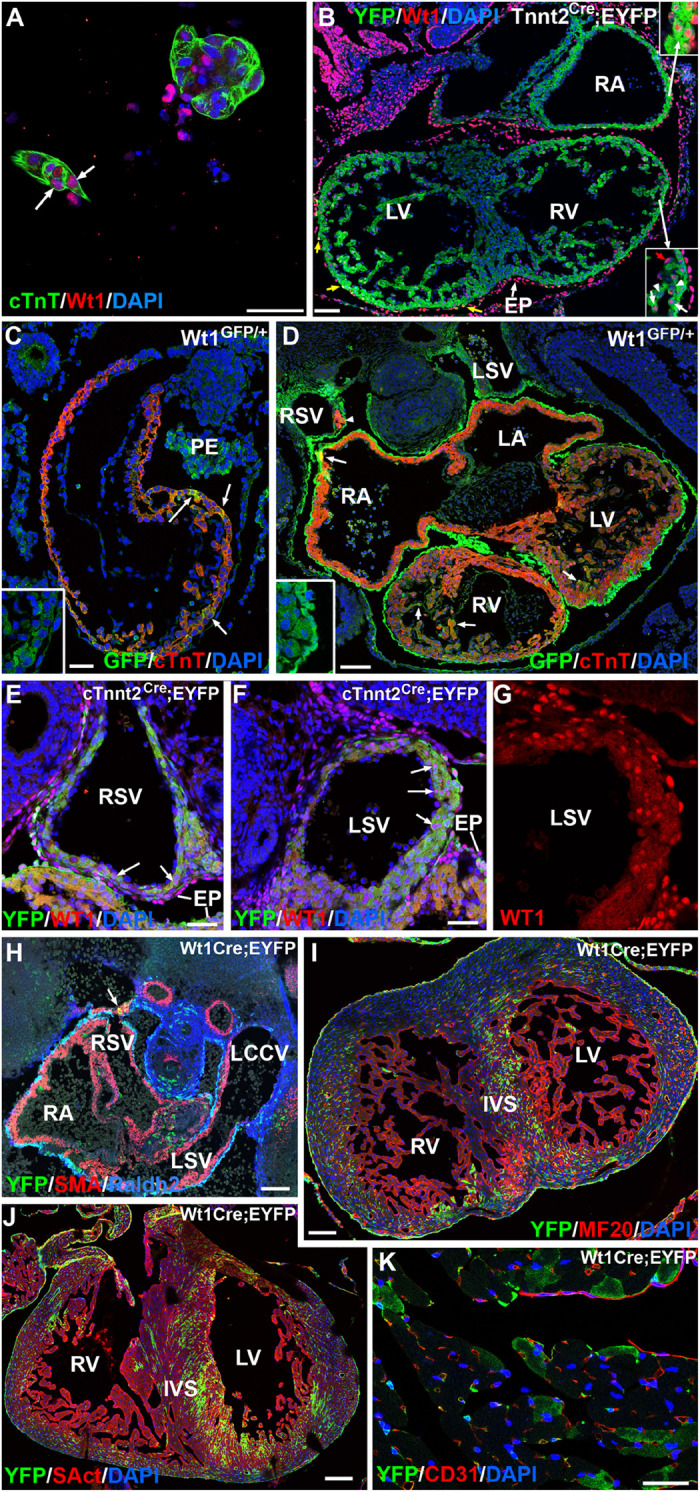
WT1 expression in cardiomyocytes. Representative images obtained from the analysis of >30 embryos. **(A)** Immunolocalization of protein WT1 in cultured cardiomyocytes characterized by cardiac troponin expression (arrow) and obtained from an E10.5 embryo. **(B)** Immunolocalization of protein WT1 in an E11.5 Tnnt2^*Cre*^;EYFP embryo. WT1 (red staining) is localized in some cardiomyocytes (inserts). Epicardial cells (EP) show expression of the protein WT1, and a few derive from a cardiac troponin-expressing lineage (yellow arrows). RA: right atrium; LV, RV: left and right ventricle, respectively. **(C,D)** WT1^*GFP/+*^ embryos, E9.5 and E11.5, respectively. Expression of the reporter reveals WT1 promoter activation in a number of cardiomyocytes of these early embryos. Colocalization with cardiac troponin (cTnT) is shown by arrows. The inserts show the separate GFP channel. Insert in **(D)** shows some GFP-expressing cardiomyocytes located in the wall of the right sinus venosus (arrowhead). LA, LV: left atrium and ventricle; PE: proepicardium; LSV, RSV: left and right sinus venosus. **(E–G)** Expression of WT1 protein in the walls of the right and left sinus venosus (RSV, LSV) of an E12.5 cTnnt2^*Cre*^;EYFP embryo. Arrows show positive nuclei of cardiomyocytes identified by the cardiac troponin lineage reporter YFP. Figure **(G)** shows a higher magnification of the left sinus venosus wall in the red (WT1) channel. A stronger expression is detected in the epicardial cells (EP). **(H–J)** Expression of the WT1 lineage reporter in the WT1^*Cre*^;EYFP line, stages 11.5, 15.5, and 19.5, respectively. Colocalization with smooth muscle actin is shown in the posterior wall of the right sinus venosus (arrow in **H**), and it becomes frequent in the ventricles in later stages, particularly in the left part of the interventricular septum (IVS). LCCV: left common cardinal vein. **(K)** Expression of the WT1 lineage reporter in an adult WT1^*Cre*^;EYFP heart. A number of cardiomyocytes show reporter expression. Scales: 50 μm, except **(D,H,I)** (100 μm) and, **(J)** (200 μm).

We further confirmed the presence of WT1 protein in cardiomyocytes by immunocytochemistry in both, cultured E10.5 embryonic cardiomyocytes and histological sections (Figures 2A,B and Supplementary Figure 3). GFP+ cardiomyocytes can be seen scattered throughout the heart in heterozygous E9.5–E11.5 Wt1^*GFP/+*^ embryos (Figures 2C,D). Expression of the reporter indicates *Wt1* promoter activation in these cTnT+ cardiomyocytes, Myocardial immunolocalization of WT1 becomes restricted to the sinus venosus in E12.5 embryos (Figures 2E–G), mainly it its posterior wall. The WT1^*Cre*^;R26R^*EYFP*^ lineage tracing system allowed us to establish the distribution of the cardiomyocyte lineage where the *Wt1* promoter was activated. As shown in Figures 2H–J, these WT1-lineage cardiomyocytes can be seen throughout the heart, with a greater abundance in the sinus venosus, left ventricle and left part of the interventricular septum. WT1-lineage cardiomyocytes can also be observed in the ventricles of adult hearts (Figure 2K).

**FIGURE 3:**
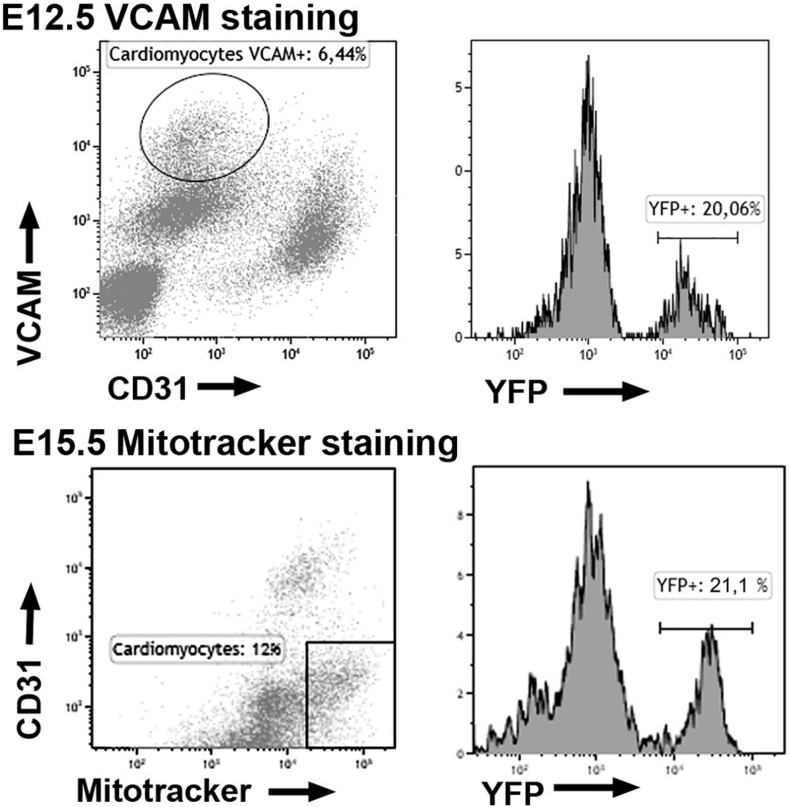
Expression of the YFP reporter in cardiomyocytes of the WT1^*Cre*^; R26R^*EYFP*^ lineage tracing line. Representative cytograms obtained from the analysis of 11 embryos. The fraction of cardiomyocytes expressing the WT1 lineage reporter (YFP+) is shown.

We quantified the frequency of WT1-lineage cardiomyocytes by analytical flow cytometry. At E12.5 and E15.5 about 18 and 25% of the cardiomyocytes show expression of the reporter, respectively (Figure 3 and Table 1). The latter percentage remains broadly constant in late embryos (not shown).

In summary, the *Wt1* promoter is transiently activated in the lineage of 20–25% of the embryonic cardiomyocytes, and WT1 protein can be detected by immunocytochemistry in a fraction of these cardiomyocytes, mainly in early developmental stages.

### Myocardial Deletion of WT1 Causes Structural Defects in the Heart

The Tnnt2^*Cre*^ driver activates Cre-mediated recombination in virtually all the myocardium at early developmental stages (Jiao et al., 2003). When we checked the efficiency of this recombination in the Tnnt2^*Cre*^;R26R^*YFP*^ model, we found that a small fraction of the epicardial cells at early stages express the YFP reporter, suggesting derivation from Tnnt2-expressing progenitors (Figure 2B). We have estimated elsewhere that about 30% of the early embryonic epicardium (E11.5) derive from a Tnnt2 expressing lineage (Carmona et al., 2020). In the present study we found that 43 ± 11% of the epicardial cells derive from a Tnnt2-expressing lineage at the stage E13.5 (mean of three embryos). Thus, the conditional deletion of WT1 in the Tnnt2 lineage should delete WT1 in all the myocardium, but only in a fraction of the epicardium and the epicardial-derived mesenchymal cells during the process of epithelial-mesenchymal transition. This fraction must be between 30–40%, since this is the percentage of Tnnt2-lineage epicardial cells when the epithelial-mesenchymal transition is at its peak (i.e., between the stages E10.5 and E13.5). We did not detect significant embryonic lethality provoked by this deletion, although the percentage of adult mutant mice significantly decreased from the expected 25% to a 13.7% The percentage of neonatal mice was also reduced (12.8%), suggesting that some mortality might occur perinatally (Table 2).

**TABLE 2:** Frequency of genotypes by age.

	Total	Wild	Heterozygotes	Mutants	% Mut
<E12.5	52	24	15	13	25.00
E13.5–E15.5	375	204	87	84	22.40
E16.5–E19.5	34	16	8	10	29.41
Neonates	39	21	13	5	12.82^†^
Adults	131	67	46	18	13.74*
Total	631	332	169	130	20.60

*Difference between the observed frequency and the expected one (25%) is significant (p < 0.05, Chi-square test). ^†^Not significant difference (p < 0.08, Chi-square test).

**FIGURE 4:**
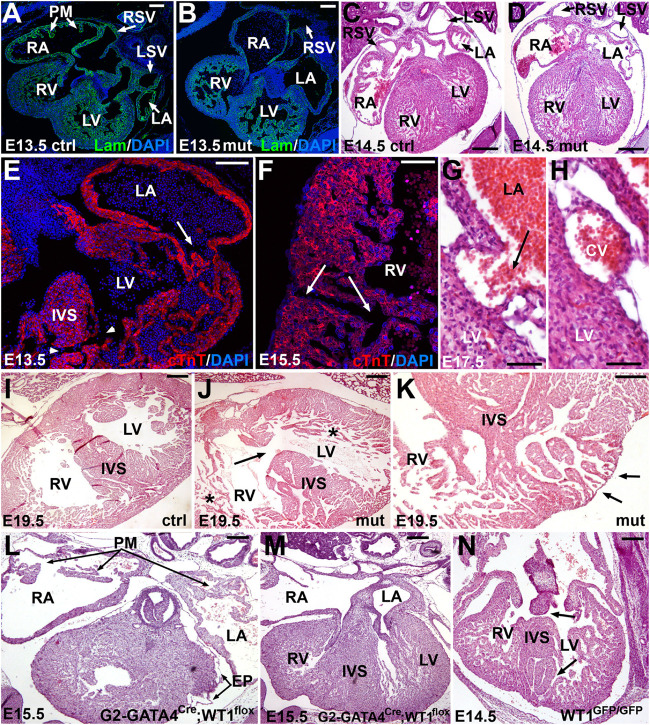
Embryonic phenotype of the conditional WT1 loss of function in cardiomyocytes (Tnnt2^*Cre*/+^; WT1^*flox/flox*^ line). Representative images obtained from the analysis of 14 mutant embryos. **(A–D)** General view of the heart in control **(A,C)** and mutant **(B,D)** embryos, stages E13.5 and E14.5. Mutant embryos show thinner ventricular walls, defective sinus venosus (shown in more detail in Figure 5) and lack of pectinate muscles (PM) in the right atrium (RA). RV: right ventricle; LA: left atrium; LV: left ventricle; RSV: right sinus venosus; LSV: left sinus venosus. **(E–H)** Defects in the myocardial walls and interventricular septum (IVS) found in mutant embryos. These defects include abnormal atrioventricular connection and muscular IVS defect (arrow and arrowheads in **E**), discontinuities in the right ventricular (RV) free wall (arrows in **F**) and connection of the left atrial (LA) lumen with a subepicardial coronary vein (CV) **(G,H)**. **(I,J)** IVS defect (arrow), thin compact myocardium and hypertrabeculation (asterisks) in an E19.5 mutant embryo **(J)** compared with a control of the same age **(I)**. **(K)** The lack of compaction of the left ventricular apex causes a diverticulum protruding in the surface of the heart (arrows). Note the lack of compact myocardium at the base of the IVS. **(L,M)** Representative E15.5 embryo of the G2-GATA4^*Cre*^;WT1^*flox*^ model of conditional deletion of WT1 in lateral mesoderm, including septum transversum and pro/epicardium (Cano et al., 2016). The main features of this phenotype are the defective sinus venosus and epicardium (EP), but the development of the pectinate muscles (PM) and the interventricular septum (IVS) are normal. **(N)** Systemic deletion of WT1 (homozygous knockin Wt1^*GFP/GFP*^, Hosen et al., 2007), E14.5, respectively. This is the most advanced stage reached by these mutant embryos. Note the defects in the IVS (arrows). Scales: 50 μm and except **(C–E)** (100 μm).

**FIGURE 5:**
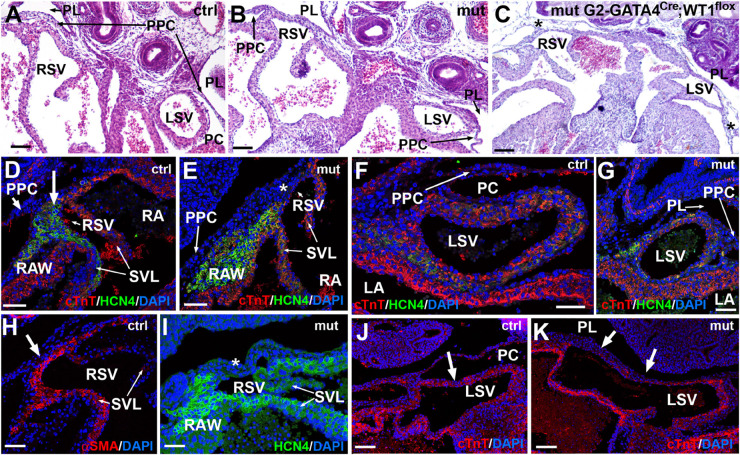
Sinus venosus defects caused by the of the conditional WT1 loss of function in cardiomyocytes (Tnnt2^*Cre*/+^; WT1^*flox/flox*^ line). **(A,B)** Comparison of the right and left sinus venosus (RSV, LSV) in E13.5 control and mutant embryos, respectively. The sinus venosus in the control embryos show a posterior wall covered by the epicardium, limited by the pericardial cavity (PC) and free from the pleuropericardial membranes (PPC). The mutant embryos show posterior walls continuous with the pleuropericardial membranes and limited by the pleural cavities (PL). **(C)** G2-GATA4^*Cre*^;WT1^*loxP*^ model of conditional deletion of WT1 in lateral mesoderm. In this E13.5 mutant embryo the PPC are lacking, and the posterior wall of the right and left sinus venosus are embedded in the body walls (asterisks). **(D–K)** Immunocytochemical features of the posterior wall of the mutant sinus venosus **(E,G,I,K)** as compared with the control embryos **(D,F,H,J)**. Stage E15.5. The mutant walls show fewer cardiomyocytes and myocardial discontinuities (asterisk in **E**, arrows in **K**, compare with arrows in **D,H,J**). Note the lack of HCN4 expression in the posterior wall of the mutant right sinus venosus (asterisks in **E,I**). The expression of HCN4 is extended in these embryos toward the right atrial wall (RAW). Scales: 50 μm.

All mutant embryonic hearts, from E13.5 onward, showed irregular shapes, thin free ventricular walls together with high degree of trabeculation, atrial pectinate muscles less developed or absent, and defective sinus venosus (Figures 4A–D, 5). In 8 out of 14 embryos analyzed (57%) we also observed structural defects in the ventricular and atrial walls (Figures 4E–K). Namely, in two embryos (14%) the atrial wall was discontinuous and communicated with the ventricular cavity (Figure 4E) or with a subepicardial vein (Figures 4G,H). Six embryos analyzed (43%) showed muscular defects in the interventricular septum (Figures 4E,J), discontinuities (Figure 4F) or diverticula in the ventricular walls (Figure 4K). Despite these myocardial abnormalities, the coronary vasculature developed normally and coronary arteries were observed in late mutant embryos and adult mutant mice (Supplementary Figure 4). The origin of the coronary arteries from the aorta showed a normal pattern in mutant mice, within the anatomical variability of this feature (Fernández et al., 2008).

We measured the ratio compact/total myocardial thickness in E13.5 and E15.5 embryos (Table 3). The ventricular compact layer was thinner in mutant embryos as compared with the total myocardial thickness. The difference was significant in the left ventricle of the E13.5 embryos and in both ventricles of the E15.5 embryos. Proliferation of cardiac cells in control and mutant E13.5 embryos was analyzed by estimating the percentage of BrdU+ cells (Table 3). The percentage of proliferating cells was significantly reduced in the mutant atria and it also showed a lower value in the ventricles, although the difference with controls was not significant.

**TABLE 3:** Proliferation index (% of BrdU+ nuclei) and ventricular compaction index (% compact layer thickness related to total wall thickness) in conditional WT1 mutant and control mice (stages E13.5 and E15.5).

		Stage E13.5		Stage E15.5	
Proliferation Iindex (%BrdU+ cells)	Atrium control	8,0.35 ± 1,0.04 (*N* = 11)	*p* < 0,0.013†		
	Atrium mutant	3,0.46 ± 1,0.10 (*N* = 5)			
	Ventricle control	5,0.45 ± 0,0.71 (*N* = 18)	*p* < 0,0.290 (n.s.)		
	Ventricle mutant	4,0.12 ± 1,0.03 (*N* = 9)			
Ventricular compaction index (% compact layer thickness related to total wall thickness)	Right ventricle control	23,0.8 ± 3,0.2 (*N* = 13)	*p* < 0,0.102 (n.s.)	58,0.4 ± 5,0.0 (*N* = 7)	*p* < 0,0.013*
	Right ventricle mutant	18,0.4 ± 1,0.5 (*N* = 19)		40,0.0 ± 3,0.9 (*N* = 7)	
	Left ventricle control	24,0.6 ± 2,0.6 (*N* = 13)	*p* < 0,0.0005*	53,0.5 ± 2,0.5 (*N* = 8)	*p* < 0,0.002*
	Left ventricle mutant	14,0.4 ± 1,0.2 (*N* = 19)		41,0.2±2,0.1 (*N* = 7)	

N is the number of sections analyzed. 6 control and 3 mutant embryos were used for the proliferation study. For the ventricular compaction index we analyzed 12 control and 18 mutant E13.5 embryos, and 5 control and 5 mutant E15.5 embryos. n.s: not significant. ^†^Mann Whitney U-test. *Student’s t-test.

We compared the phenotype of the deletion of WT1 in the cardiac troponin lineage with that from other two murine models of WT1 loss of function, a lateral mesoderm/proepicardial/epicardial conditional deletion (G2-GATA4^*Cre*^;WT1^*flox*^, Cano et al., 2016) and a systemic deletion in all the embryonic tissues (homozygous knockin WT1^*GFP/GFP*^, Hosen et al., 2007). Both deletions are lethal at midgestation. The heart with pro/epicardial deletion of WT1 showed lack of coronary arteries, but normal development of the interventricular septum and pectinate muscles (Figures 4L,M). Free myocardial walls never showed discontinuities. However, the embryos with systemic WT1 loss of function showed defects in the interventricular septum similar to those displayed by the Tnnt2^*Cre*^;WT1^*flox*^ mutant embryos (Figure 4N).

As stated above, both sinus venosus horns were abnormal in the mutant embryos (Figure 5). The left sinus venosus was embedded into the left pleuropericardial membrane, while the right sinus was also abnormally connected with the right pleuropericardial membrane. In both cases, the posterior walls of the sinus horns in mutants were limited by the pleural cavities, while the sinus horns in control mice were surrounded by the pericardial cavity (Figures 5A,B). The right sinus horn lacked myocardium in its posterior part (asterisks in Figure 5E), a region that is muscular in the controls of the same stage (Figures 5D,H). Due to the lack of muscle in this area, most of the HCN4 expression was localized in the wall of the right atrium (Figures 5E,I). This abnormal pattern of HCN4 expression was also observed in the area corresponding to the sinoatrial node in E18.5 embryos, an area that was much smaller in mutants (Supplementary Figure 5). The myocardial layer was very thin on the posterior part of the left sinus venosus (Figures 5G,K). Both, the systemic deletion of WT1 (Norden et al., 2010) and the lateral mesoderm/proepicardial/epicardial-specific deletion of WT1 using the G2 enhancer of the GATA4 gene (Cano et al., 2016) showed a more severe phenotype, with lack of pleuropericardial membranes and complete loss of the posterior walls of the sinus venosus, which does not form clearly defined horns (asterisk in Figure 5C).

### Adult Mice With Myocardial Deletion of WT1 Show Anomalies in Cardiac Structure and Function

**FIGURE 6:**
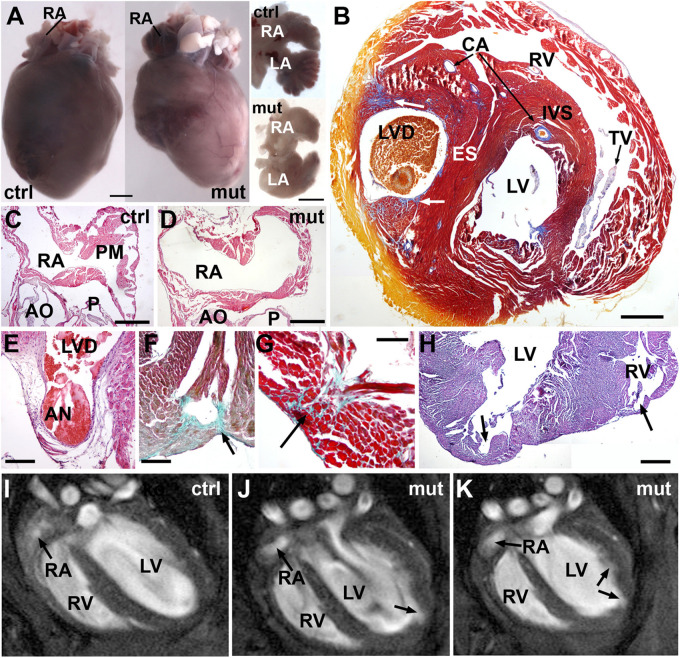
Adult phenotype of the conditional WT1 loss of function in cardiomyocytes (Tnnt2^*Cre*/+^; WT1^*flox/flox*^ line). Representative images obtained from the analysis of 9 mutant mice. **(A)** Representative example of a mutant heart compared with a control. The mutant heart is smaller and shows irregular profiles. The dissected atria (ventral view) show different shapes due to lack of pectinate muscles. **(B)** Mallory’s trichrome stain. This heart of an adult mutant mouse shows an ectopic septum (ES) partially dividing the left ventricle in two chambers, the left one forming a blind diverticulum (LVD). Note some areas of fibrosis (arrows). Coronary arteries (CA) are well developed. IVS: interventricular septum; TV: tricuspid valve. **(C,D)** Right atrium (RA) of a control and a mutant adult heart, H&E stain. Note the lack of pectinate muscles (PM) in the mutant atrium. AO, aortic root; P, pulmonary root. **(E)** The left ventricle diverticulum shown in **(B)** ends in a fibrous aneurism (AN) located in the base of the left ventricle. H&E stain. **(F–H)** Defects in the free ventricular walls consisting of disorganized and fibrotic areas (arrows in **F,G**, Masson-Goldner’s stain) and diverticula (arrows in **H**, H&E stain). **(I–K)** MRI images of the heart in a control **(I)** and a mutant mouse **(J,K)**. The inner surface of the left ventricle shows an irregular surface with an apical diverticulum (arrow). Note the globose shape of the left ventricle and the rounded right atrium (RA) lacking of pectinate muscles. A video of the RMI images is available as Supplementary Material. Scales: **(A)**: 1 mm; **(B–D)**: 0.5 mm; **(E–H)**: 250 μm.

As stated above, mutant mice are viable although their frequency is about a half of the expected in the adult population. In adult mutant mice, the heart weight with respect to total body weight was significantly reduced (0.54 ± 0.06% in mutants vs. 0.84 ± 0.11% in controls, *N* = 9 and 6, respectively, *p* < 0.05). The heart showed a highly irregular shape (Figure 6A), lacking pectinate muscles in the right atrium, which appeared round-shaped (Figures 6A,C,D). In a case, the left ventricle was partially divided by an ectopic muscular septum (Figure 6B). One of the left ventricular cavities ended in an aneurism protruding in the base of the left ventricle (Figure 6E). Areas of fibrosis, disorganization of the myocardium and diverticula were frequently observed in the cardiac walls (Figures 6B,F–H). *In vivo* MRI images confirmed the anomalies of the right atrium and the left ventricular wall [Figures 6I–K and Supplementary Videos 1 (control) and 2 (mutant)].

Adult mutant mice also showed alterations in the ECG, with significant increase of the RR interval and decrease of the PR interval as compared to controls (*p* < 0.05, Student’s *t*-test). The QRS interval was also significantly longer in the mutants (16.3 vs. 14.4 ms in the controls, *p* < 0.05). On the other hand, the amplitudes of the P and R waves, and the height of the S-T interval (corresponding to the J wave, see section “Discussion”) were significantly reduced in mutant mice (Table 4 and Supplementary Figure 6).

**TABLE 4:** ECG parameters in conditional WT1 mutant and control adult mice.

	RR Interval (ms)	Heart Rate (BPM)	PR Interval (ms)	P Duration (ms)	QRS Interval (ms)	QT Interval (ms)	QTc (ms)	JT Interval (ms)	Tpeak Tend Int. (ms)	P Amplitude (μV)	Q Amplitude (μV)	R Amplitude (μV)	S Amplitude (μV)	ST Height (μV)	T Amplitude (μV)
Control (*N* = 20)	256.6 ± 13.3	248.1 ± 10.3	39.6 ± 0.8	14.9 ± 0.5	14.4 ± 0.6	32.7 ± 0.9	66.2 ± 3.2	18.0 ± 0.8	11.8 ± 0.7	50.2 ± 6.9	9.75 ± 1.3	407.7 ± 16.3	9.2 ± 13.7	69.6 ± 10.3	120.3 ± 9.6
Mutant (*N* = 24)	304.0 ± 15.1	216.0 ± 13.4	36.8 ± 1.1	15.6 ± 0.5	16.3 ± 0.5	33.5 ± 1.0	62.8 ± 2.3	16.7 ± 1.2	10.9 ± 0.8	23.0 ± 9.9	8.94 ± 1.1	323.8 ± 17.4	50.9 ± 18.1	0.59 ± 15.0	97.5 ± 10.7
Student’s *t*-test	0.026*	0.104	0.045*	0.361	0.024*	0.552	0.383	0.397	0.452	0.034*	0.628	0.001**	0.014*	0.001**	0.127

Mean ± s.e.m. **p < 0.01, *p < 0.05.

### Transcriptomic Analysis of the Mutant Hearts Reveals Alterations in the Expression of Genes Related to Myocardial Function

**FIGURE 7:**
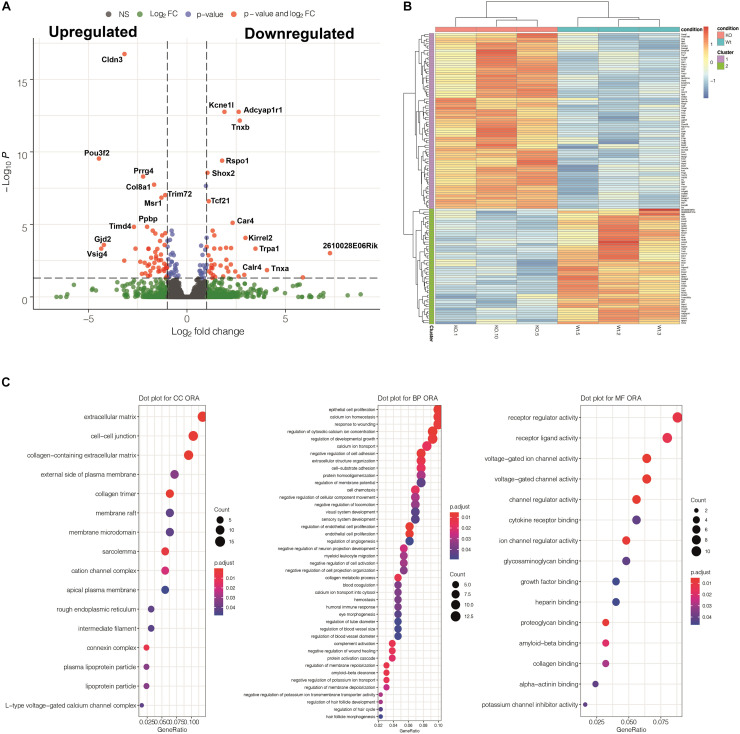
Summary of the results obtained from a transcriptomic comparison between three Tnnt2^*Cre*/+^; WT1^*flox/flox*^ mutant and three control embryos (stage E13.5). **(A)** Volcano plot showing differentially expressed genes (DEGs) detected by DESeq2. **(B)** Heatmap showing a cluster analysis of DEGs based on the K-means method. **(C)** Gene Ontology Analysis implemented through over representation analysis (ORA) test for molecular function, biological process and cellular component categories. Regulation of calcium and voltage-gated ion channels are among the most overrepresented biological processes and molecular functions affected by the WT1 loss of function in the Tnnt2 lineage. Genes with significant *p*-Values are detailed in Supplementary Table 3.

RNASeq analysis was performed on whole E13.5 hearts. This is the stage where the main morphological features of the mutant phenotype first appear. A total number of 137 genes were differentially expressed in the mutant heart as compared with controls (adjusted *p*-values <0.05). 80 of them were upregulated and 57 were downregulated (Supplementary Table 3 and Figure 7). GO functional enrichment analysis performed through over representation analysis method suggested that the calcium ion regulation was deeply altered in the mutant heart. In fact, three of the six main biological processes affected by the WT1 loss of function were associated to gene sets related with calcium ion homeostasis, regulation of cytosolic calcium concentration and calcium ion transport. On the other hand, voltage-gated channel activities were among the most over-represented molecular functions altered in mutant hearts. Interestingly, expression of epicardial genes related with myocardial proliferation (*Aldh1a2*, *Igf2*, Brade et al., 2011) did not significantly change in mutant hearts.

**FIGURE 8:**
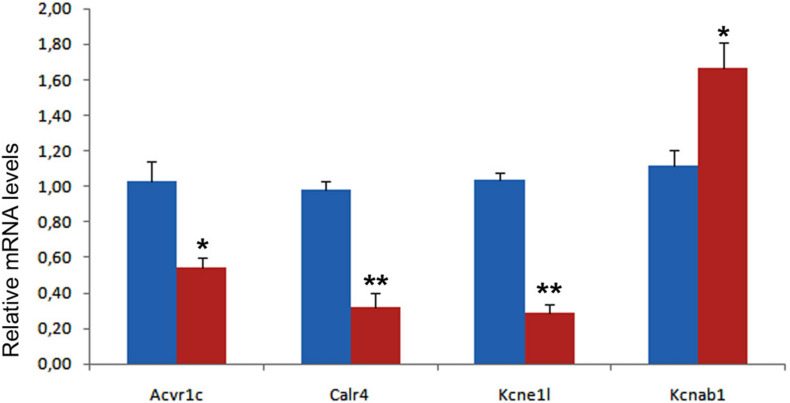
Validation of four differentially expressed genes in control (blue) and mutant (red) hearts from E13.5 embryos. Each PCR reaction was carried out in triplicate and repeated in at least three distinct pooled biological samples. **p < 0.01, *p < 0.05, Student’s *t*-test.

We have validated by qPCR a number of differentially expressed genes that can be related with the observed phenotype (Figure 8). A calcium-handling protein, calreticulin-4, and an ancillary subunit of voltage-gated potassium channels, Kcne1l (*aka* Kcne5) were significantly down-regulated (see also Supplementary Figure 7 for immunolocalization of Calr4). The type I Activin/Nodal receptor Acvr1c (*aka* ALK7) was also significantly down-regulated in mutant hearts. In contrast, the potassium channel subunit Kcnab1 (Kvβ1) a modulator of voltage-gated K + (Kv1) channel function in mouse ventricular myocytes (Aimond et al., 2005), was significantly upregulated.

## Discussion

The expression of WT1 in the embryonic epicardium is essential for the development of the heart. Both, systemic or epicardial-specific loss of function of WT1 results in defective generation of EPDC, abnormal cardiac vascularization and thinning of the ventricular myocardium, provoking the death of the mouse embryos by midgestation (Martínez-Estrada et al., 2010). This embryonic lethality in both, systemic and epicardial WT1 deletion, could have masked a cell-autonomous function of WT1 in the myocardium.

Our results show that a part of the embryonic cardiomyocytes express WT1 at low levels as suggested by previous reports (Villa del Campo et al., 2016; Velecela et al., 2019; Van Eif et al., 2020; Wagner et al., 2021). This is supported by evidence from the reporter mice (knockin GFP), the WT1 lineage tracing system (Wt1^*Cre*^;R26R^*EYFP*^) and the immunohistochemical detection of WT1 protein, which was only possible in early stages, when WT1 expression levels are relatively higher. Detectable WT1 protein persisted by midgestation in the sinus venosus, but it was not traceable by immunohistochemistry in the atrial or ventricular myocardium after the stage E12.5. However, a recent report showed a speckled expression in some cardiomyocytes of later embryonic stages, using a sensitive immunohistochemistry approach (Wagner et al., 2021). The speckled pattern suggests that the Wt1(+KTS) isoform is expressed in these cells (Herzer et al., 2001).

The embryonic origin of the WT1-lineage derived cardiomyocytes is unclear. The former proposal of an epicardial origin of a set of cardiomyocytes (Cai et al., 2008; Zhou et al., 2008) was challenged by the evidence of expression of WT1 in cardiomyocytes before the emergence of the epicardium/epicardial-derived cells (Rudat and Kispert, 2012; Villa del Campo et al., 2016; see also Figure 2C). An interesting possibility has been raised by the recent report of Tyser et al. (2021) describing the results of single-cell RNA sequencing on the microdissected cardiac region of mouse embryos, from early cardiac crescent to linear heart tube stages. These authors identified a population of progenitor cells (named as the juxta-cardiac field, JCF) that can contribute to both, cardiomyocytes and proepicardium. The distribution of the cardiomyocytes derived from the JCF is noticeably similar to that found by us for the WT1-lineage cardiomyocytes and extends over the four cardiac chambers. Using the web interface provided by the authors^3^ we have found that WT1 is expressed in 31% of the cells included in the cluster Me5, corresponding to the JCF and characterized by a low Nkx2.5 expression. It seems likely that at least a part of the WT1-lineage cardiomyocytes derive from these common cardiogenic/epicardial progenitors.

Our conditional deletion of WT1 in the Tnnt2 lineage should have knocked down the transient WT1 expression specifically in the myocardium. However, when we checked the recombination induced by the Tnnt2^*Cre*^ driver in Tnnt2^*Cre*^;EYFP mice, we found reporter expression in a part of the epicardium. This fact is probably due to an early activation of the Tnnt2 promoter in common epicardial and myocardial progenitors located in the secondary heart field or even in the recently described juxta-cardiac field mentioned above. This epicardial deletion was partial and in fact it did not affect to most of the early epicardium. Consequently, the early generation of EPDCs should not be significantly disturbed in our embryos. We cannot completely rule out that some phenotypic features observed in the mutant embryos can be due to the loss of function of Wt1 in a fraction of the epicardium, particularly the thinning of the compact ventricular myocardium. However, the impact of the hypomorphic mutation of WT1 in the epicardium should be minor for a number of reasons: (1) Coronary development, a process critically dependent on epicardial-derived cells, was normal in the mutant embryos; (2) Proliferation index in the ventricles by E13.5 was reduced in mutant embryos, but the difference with controls was not statistically significant; (3) Expression of *Aldh1a2*, and *Igf2*, strongly reduced in epicardial WT1 loss-of-function (Brade et al., 2011), showed no changes in our RNASeq analysis, suggesting that the signaling role of the epicardium, where Igf2 plays a key role (Li et al., 2011) is maintained in the mutant embryos; and (4) The proepicardial and epicardial deletion of WT1 (G2-GATA4^*Cre*^ model) does not show features such as lack of pectinate muscles or interventricular septum/free wall defects.

Thus, most of the phenotypic features observed in our mutants can be attributed to a cell autonomous function of Wt1 in cardiomyocytes. For example, the defects observed in the interventricular septum, the disorganization and diverticula in the atrial and ventricular walls, the anomalous development of the sinus venosus and the right atrium pectinate muscles, and the electrocardiographic alterations, as discussed below.

Our conditional deletion of WT1 in the Tnnt2 lineage provoked a defect in the ventricular compaction, leading to an excess of trabeculation. This abnormal compaction can explain the defects in the interventricular septum, the discontinuities and disorganization observed in the free ventricular walls, and also the diverticula and aneurisms observed in some mutant embryos and adults. The fibrotic areas observed in adults would be the consequence of repairing processes in these damaged areas. In humans, left ventricular non-compaction appears more associated to ventricular septal defects than to any other congenital heart disease (Costa-Marques et al., 2020). On the other hand, congenital ventricular aneurisms and diverticula are rare cardiac malformations in humans (Ohlow et al., 2015). The pathogenesis of these malformations is mostly unknown, but defects in structural sarcomeric proteins can be involved. In fact, downregulation of cardiac troponin in chick embryos results in left ventricular diverticula (Ohlow, 2017). However, our RNASeq analysis suggests that the observed defects might be more related to cardiomyocyte physiology than to structural proteins as discussed below.

A failure to form myocardialize sinus venosus horns had been described in the systemic WT1 mutant mice and in the lateral mesoderm/epicardial-specific deletion of WT1 (Norden et al., 2010; Cano et al., 2016). This defect had been attributed to the expression of WT1 in the pleuropericardial membranes and the mesenchyme surrounding the cardinal veins (Norden et al., 2010). We have observed a milder phenotype in our embryos, with defective myocardialization of the posterior walls of the sinus horns, which are abnormally connected to the pleuropericardial membranes and are not surrounded by epicardium. Since the pleuropericardial membranes do not derive from a Tnnt2-expressing lineage, we can conclude that the defect observed in the sinus venosus of our mutant embryos is due to the lack of expression of WT1 in the myocardium from this cardiac region. In fact, this is the myocardial tissue where WT1 protein can be immunolocalized for a longer period. It is uncertain if anomalies in the sinus venosus walls, including the abnormal expression pattern of the sinoatrial node marker HCN4 (see Figures 5E,I) could be related with the longer RR intervals detected in mutant mice.

An intriguing feature of the WT1 loss of function in the Tnnt2-expressing lineage is the lack of pectinate muscles and the abnormal shape of the atria in both mutant embryos and adults. A similar atrial phenotype (hypoplastic atria and lack of pectinate muscles) has been described in a model of myocardial deletion of Mib1, a key element for the Notch signaling pathway (Captur et al., 2016). Myocardial-specific Mib1 deletion also provokes thin ventricular myocardium and excessive trabeculation, a phenotype described as an animal model of left ventricular non-compaction (Luxán et al., 2013). Interestingly, another model of Notch inhibition based in Cre-inducible expression of a dominant-negative truncated form of mastermind-like protein shows anomalous atrioventricular node development. These mice are viable and their cardiac morphology seems normal, but their electrocardiogram displays a shorter P-R interval indicative of a disruption of the AV nodal delay (Rentschler et al., 2011). We have also observed a significant shortening in the P-R interval in our adult mutant mice. Thus, the close similarities between our model of WT1 loss of function in myocardium and these models of cardiac inhibition of Notch signaling might suggest some connection between the WT1 loss of function and the Notch pathway in cardiomyocytes. However, our RNASeq analysis (see below) did not reveal significant changes in the components of the Notch pathway in the mutant embryos, and this hypothetical relationship with WT1 function will need of further investigation.

Transcriptomic analysis of the E13.5 mutant hearts shows that the calcium homeostasis, regulation and transport are altered after deletion of WT1. These three processes are between the six most relevant according to the gene ontology-based overrepresentation analysis. Calreticulin-4 (Calr4) is repressed in the mutants as confirmed by qPCR and supported by immunolocalization of the protein (see Supplementary Figure 7). Calr4 is a calcium-binding/storage protein localized in the endoplasmic reticulum (ER), where it acts as a chaperone preventing the exportation of misfolded proteins from ER to the Golgi apparatus. This protein is highly expressed in the developing myocardium and it is essential for cardiac development (Michalak et al., 2002). Importantly, mice deficient for Calr4 show decreased ventricular wall thickness and hypertrabeculation, a phenotype very similar to that shown by our mutants (Mesaeli et al., 1999; Rauch et al., 2000). Lozyk et al. (2006) also described a downregulation of N-cadherin in cardiomyocytes of calreticulin-deficient mouse embryos. This observation could be related with the defective compaction of the cardiomyocytes in our mutants, given the crucial role played by N-cadherin in cardiomyocyte adhesion and ventricular trabeculation (Radice et al., 1997; Miao et al., 2019).

WT1 itself has also been considered as an important transcription factor involved in Ca^2+^ homeostasis (Ritchie et al., 2011). This factor negatively regulates STIM1, an ER Ca^2+^ sensor and activator of store-operated Ca^2+^ entry in the cytosol, by binding to the EGR1 response elements of the *STIM1* promoter (Ritchie et al., 2010). STIM1 is expressed in the sinoatrial node (Zhang et al., 2015). STIM1 knockdown in cardiomyocytes causes SAN dysfunction, reduction in heart rate, arrhythmias and lethality in adult mice (Ohba et al., 2009; Zhang et al., 2015) while STIM1 overexpression in the heart leads to aberrant calcium handling and lethal cardiomyopathy (Correll et al., 2015). Thus, WT1 can be playing a role for Ca^2+^ homeostasis in embryonic cardiomyocytes, and altered Ca^2+^ handling in these cells can cause structural defects in the myocardium perhaps due to dysregulation of adhesion proteins.

We have discussed above how developmental defects in sinus venosus and right atrium could be related with the longer R-R and the shorter P-R intervals observed in the mutant mice. Adult mutant mice also showed a longer QRS duration (Table 4), suggesting anomalies in the ventricular conduction system. We have observed that the amplitude of the P and R waves, and the height of the S-T interval, are significantly lower in mutant mice. The mice ECG actually lacks of an isoelectric S-T segment, which is replaced by the J wave, which represents the early repolarization of the ventricles (Boukens et al., 2014). These differences in wave amplitude could be related with the irregular shape and the small size of the mutant heart. However, the differences in the duration of the intervals can be rather explained by the observed changes in expression of ionic channels.

The Kcne1l (=Kcne5) and the Kcnab1/Kvb1 regulatory subunits of voltage-gated potassium channels are downregulated and upregulated in the mutant heart, respectively. Both are important regulators of the action potential and their alterations have been related with changes in QRS interval duration (Aimond et al., 2005; Ohno et al., 2011; Palmer et al., 2012; Tur et al., 2016). Human KCNE5 mutations are associated with atrial fibrillation and Brugada syndrome, and they may predispose to cardiac arrhythmias (Abbott, 2016). We have found that Kcne1l is strongly downregulated in the WT1-mutant myocardium. Kcne1l deletion in mice provokes ventricular premature beats and increased susceptibility to induction of ventricular tachycardia (David et al., 2019). Finally, another down-regulated gene in our transcriptomic analysis, the nodal receptor *Acvrc1/ALK7*, is also related with the electrophysiology of the heart. Mice deficient for ALK7 are viable, but they show prolonged repolarization and increased predisposition to ventricular arrhythmias. Ying et al. (2016) showed that ALK7 mediated signaling is essential for maintaining repolarizing K^+^ currents in ventricular cardiomyocytes. Thus, differentially expressed myocardial genes in E13.5 embryos might be related to the observed electrocardiographic alterations in the adult mutant mice, although it would be necessary to check if the expression differences are maintained in adults.

In summary, we have confirmed a transient expression of WT1 in a population of embryonic cardiomyocytes, and we have shown that this myocardial expression, and not only the well-characterized epicardial WT1 expression, is required for normal cardiac development.

## Data Availability Statement

The data presented in the study are deposited in the University of Malaga Repository (https://dx.doi.org/10.24310/riuma.22437). RNASeq data are available at Gene Expression Omnibus repository (accession number GSE178220).

## Ethics Statement

The animal study was reviewed and approved by Committee on Ethics of Animal Experiments of the University of Málaga (procedure code 2018-0018).

## Author Contributions

SD, SB, and FH-T: performing experiments and collecting data. JG and MJ-N: collecting data. FH-T, AA, DF, MJ-N, RM-C, and RC: data analysis and interpretation. RM-C and RC: research design and manuscript writing. MJ-N, RM-C, and RC: final manuscript approval. All authors contributed to the article and approved the submitted version.

## Conflict of Interest

The authors declare that the research was conducted in the absence of any commercial or financial relationships that could be construed as a potential conflict of interest.

## Footnotes

1 https://en.novogene.com/

2 http://bioconductor.org

3 https://marionilab.cruk.cam.ac.uk/heartAtlas/
