# Supplementary material for: Deletion of the Wilms’ Tumor Suppressor Gene in the Cardiac Troponin-T Lineage Reveals Novel Functions of WT1 in Heart Development
Source: Front Cell Dev Biol. 2021 Jul 22;9:683861. doi: 10.3389/fcell.2021.683861 (PMC8339973; doi:10.3389/fcell.2021.683861)
Supplement: Supplementary file 1 [file Data_Sheet_1.docx]

Supplementary Material


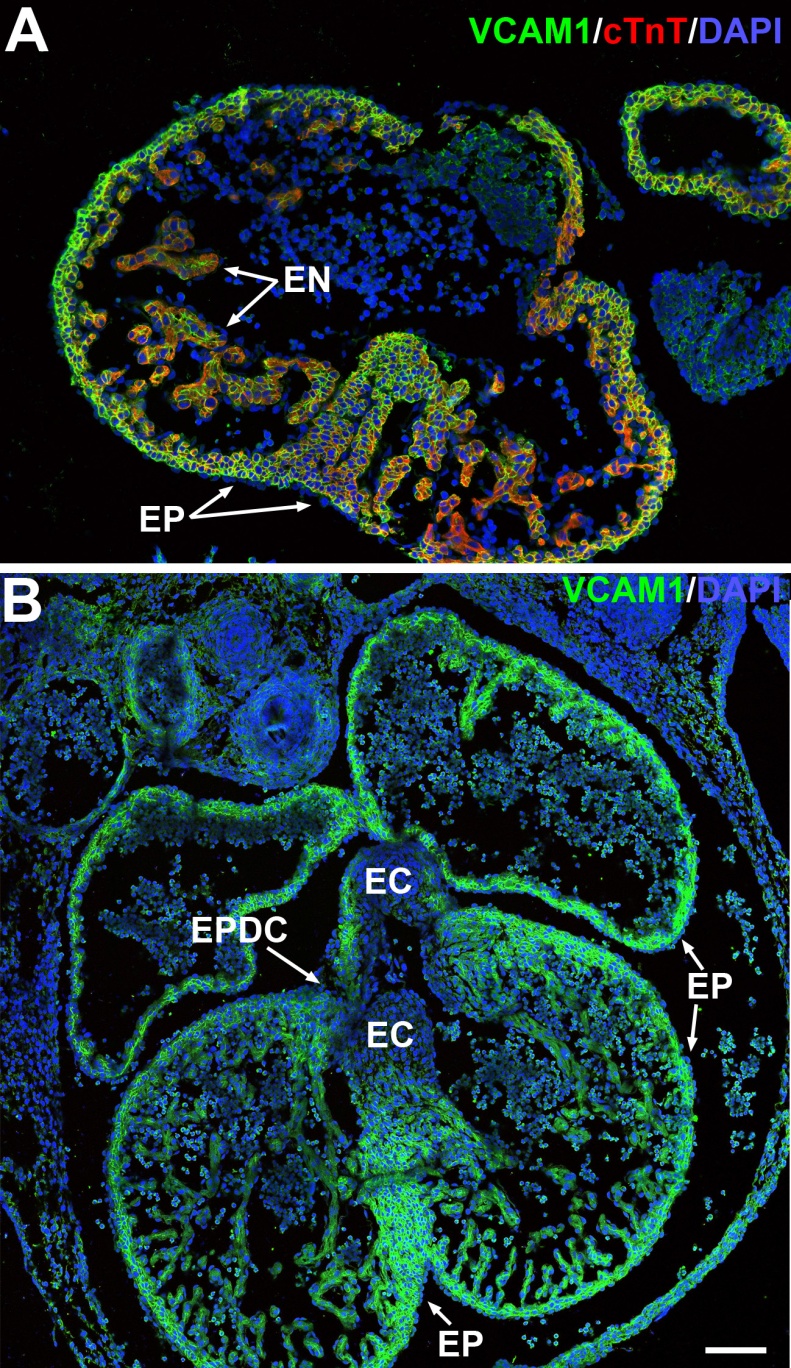


**Supplementary figure 1:** VCAM1 expression in the heart of E11.5 (A) and E12.5 (B) mouse embryos. VCAM1 is expressed mainly in cardiomyocytes by these stages. Epicardium (EP), endocardium (EN) and most of the early epicardial-derived cells (EPDC) and endocardial cushion cells (EC) are VCAM1-negative.

**
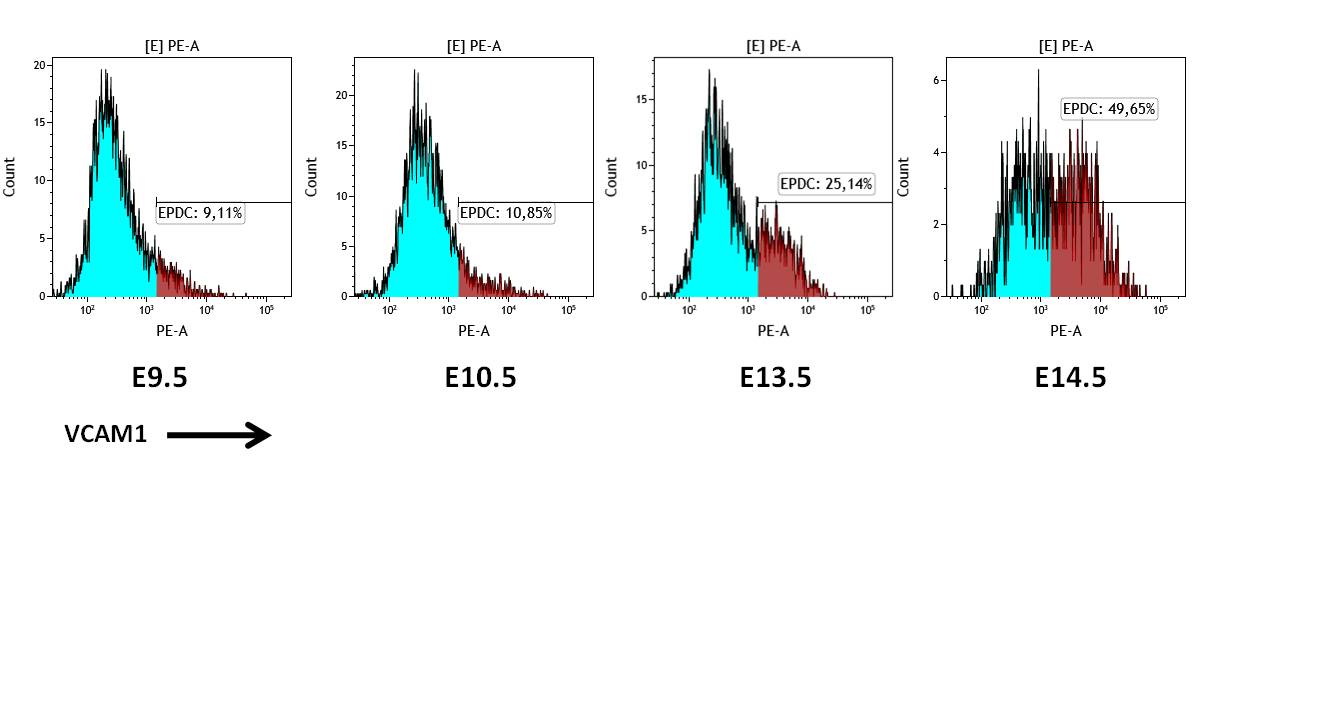
Supplementary figure 2: Expression of VCAM1 in the GFP^high^ population from WT1^GFP/+^ knockin embryos.** VCAM1 expression progressively increases in epicardial-derived cells (EPDC) as the epithelial-mesenchymal transition of the epicardium advances.

**
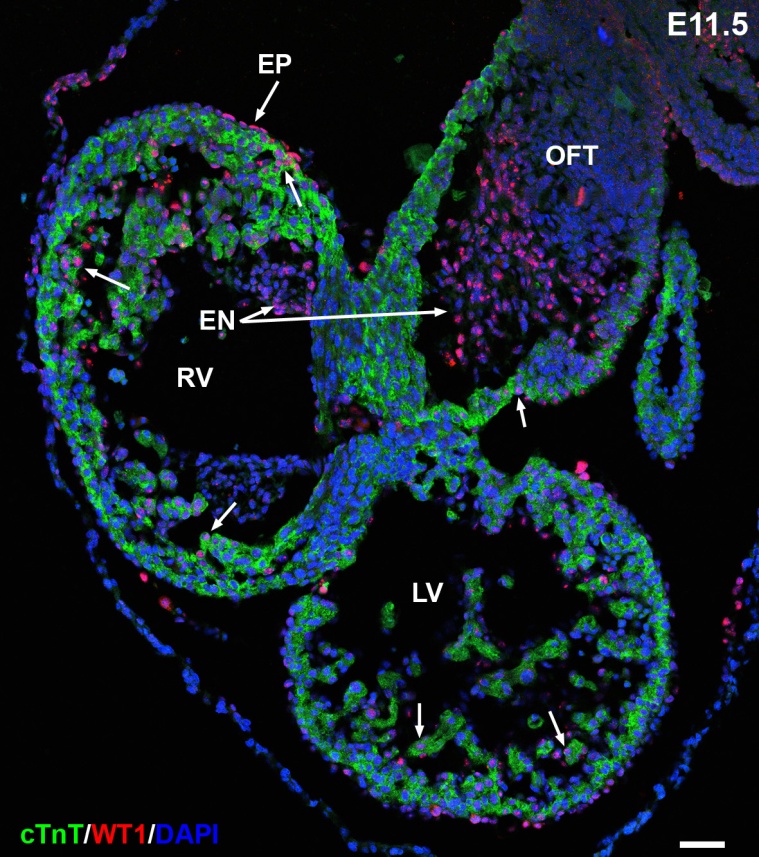
**

**Supplementary figure 3: Immunolocalization of WT1 in cTnT-expressing cardiomyocytes (E11.5).** WT1-positive nuclei (arrows) can be seen in cardiomyocytes scattered throughout right and left ventricles (RV, LV) and outflow tract (OFT). A portion of the epicardial (EP) and endocardial (EN) cells are also expressing WT1.


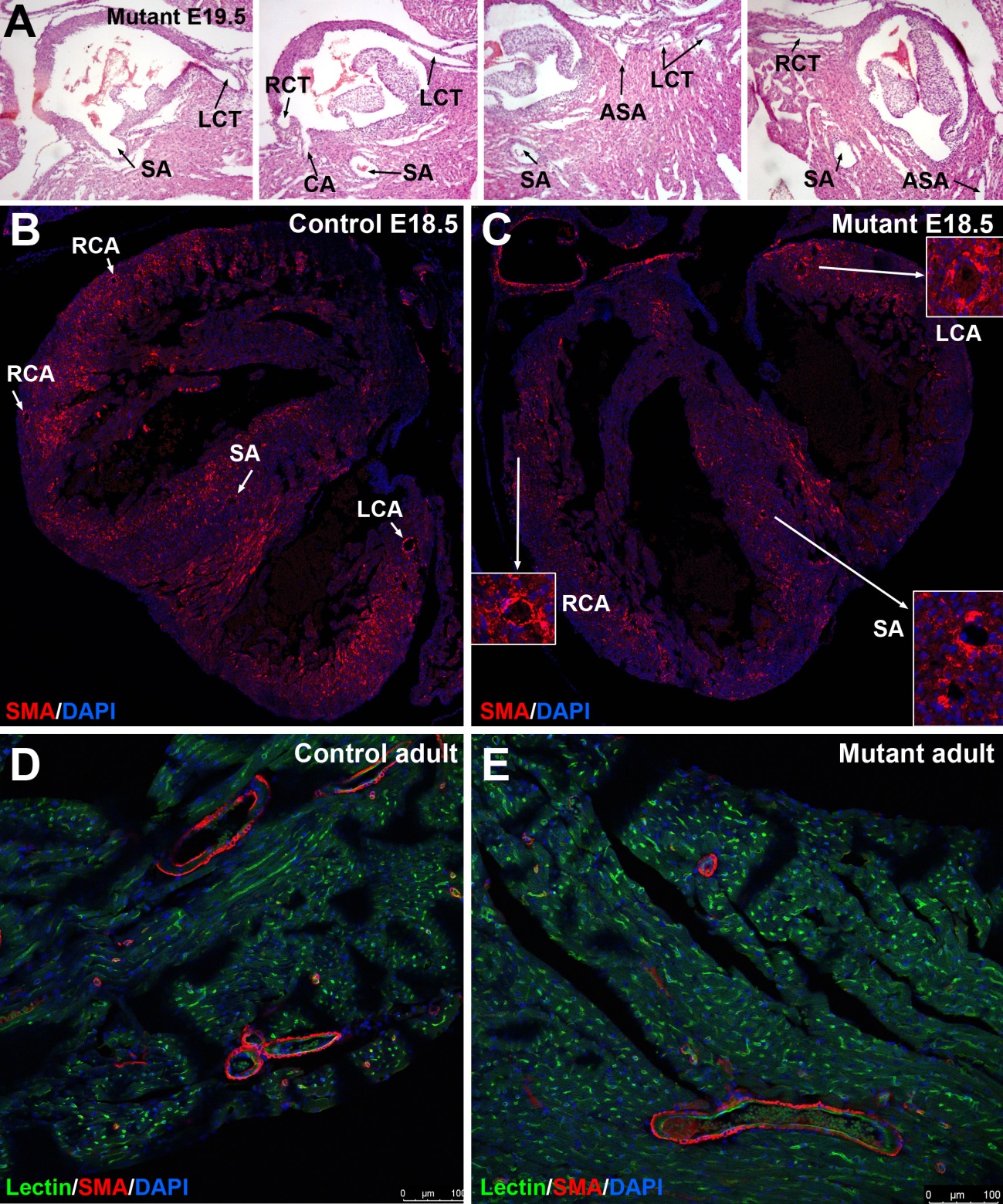


**Supplementary figure 4. Coronary vascularization in control and mutant (**Tnnt2^Cre^; WT1^fl/fl)^ **embryos:** **A**: Origin of the main coronary arteries in an E19.5 mutant embryo. Serial sections stained with H&E. The main septal artery (SA) and the conal artery (CA) arise independently from the right aortic sinus. Left and right coronary trunks (LCT, RCT) emerge from the corresponding aortic sinuses. The left coronary trunk gives rise to an accessory septal artery (ASA). This anatomical pattern is usual in mice (Fernández et al., 2008). **B-D.** Coronary vascularization is normal in E18.5 embryos (B,C) and in adult (D,E) mutant Tnnt2^Cre^; WT1^fl/fl^ mice. RCA, LCA: right and left coronary arteries.


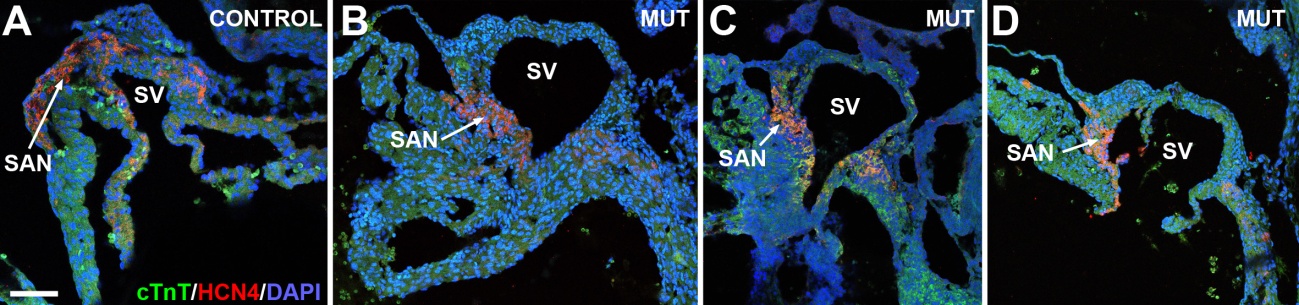


**Supplementary figure 5. HCN4 expression in the region of the sinoatrial node (SAN) from a control (A) and a mutant embryo (B-D, consecutive sections), stage E18.5**. The area expressing HCN4 is much smaller in the mutant embryo. SV: remains of the right sinus venosus incorporated to the right atrium.

**
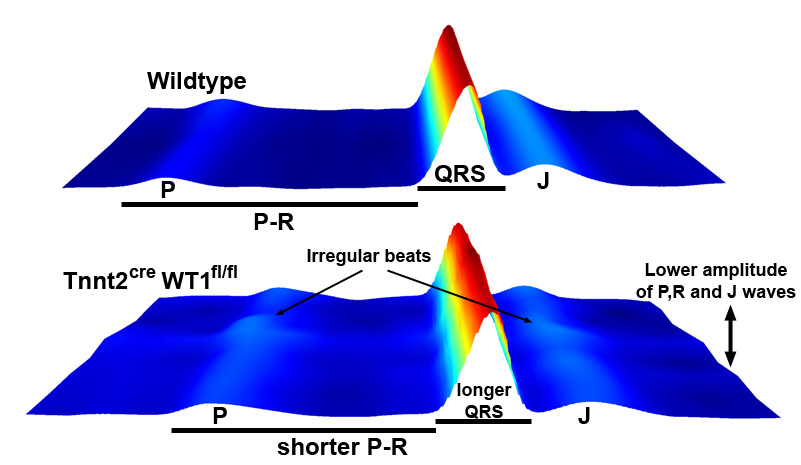
**

**Supplementary figure 6.** Waterfall plot obtained from representative ECGs of a control and a mutant adult mouse (Tnnt2^Cre^; WT1^fl/fl^ line). The main features of the mutant ECG are the shortening of the P-R interval, a longer QRS interval and lower P, R and J waves, as indicated on the figure. Note the irregular beats in the mutant plot.

**
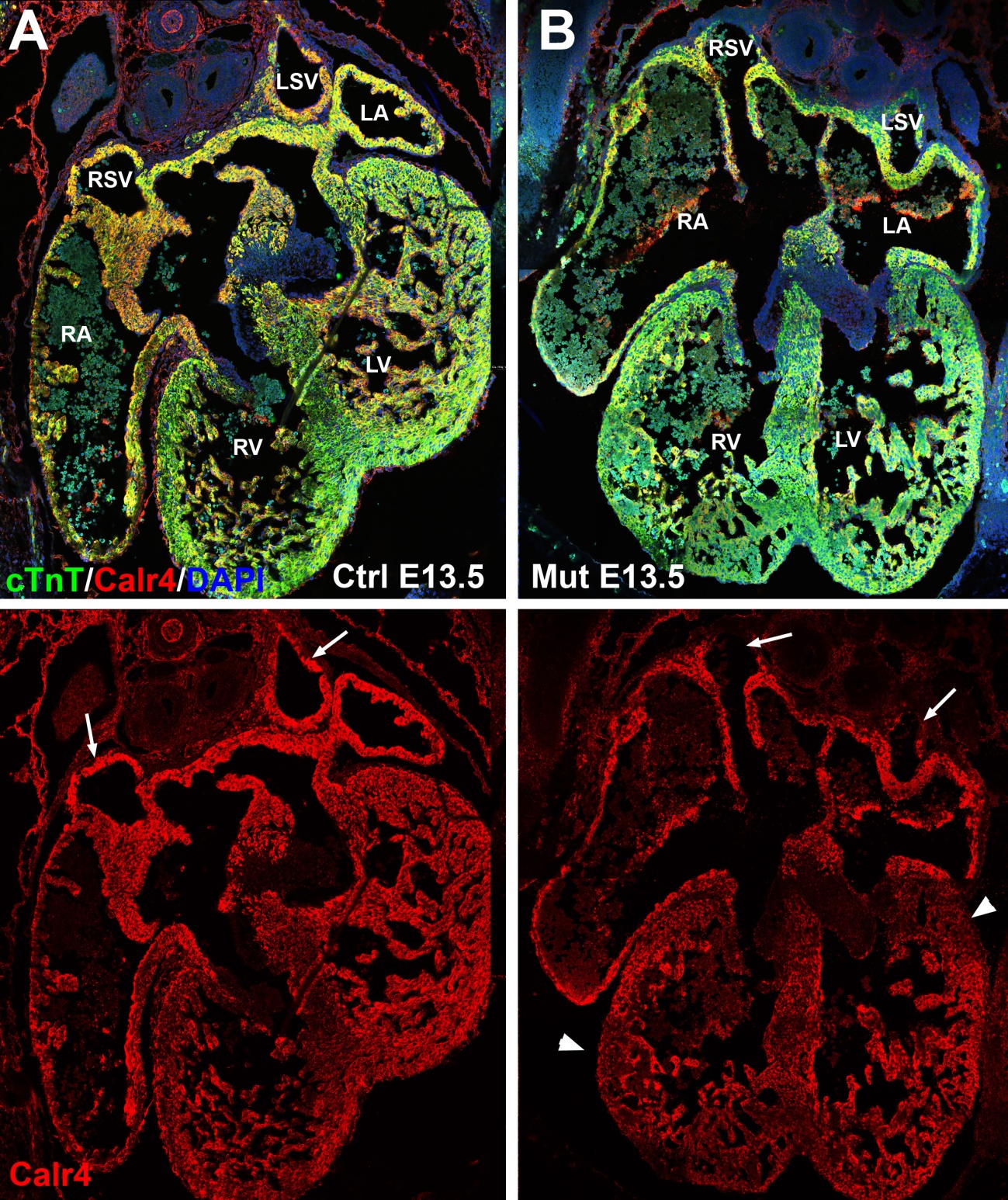
**

**Supplementary figure 7.** Calreticulin-4 expression in control (A) and mutant (B) E13.5 embryos. The Calr4 channel is shown in the bottom images. The mutant lacks of Calr4 expression in the posterior wall of the right and left sinus venosus (RSV, LSV, arrows) and it also shows patches of reduced expression in the right and left ventricles (RV, LV, arrowheads). RA and LA: right and left atrium.

**Supplementary table 1:** Antibodies

| Antibody | Supplier | Catalog | Dilution |
| --- | --- | --- | --- |
| Mouse monoclonal anti-VCAM-1 PE | Thermo Fisher | 12-1061-80 | 1/100 |
| Mouse monoclonal anti-CD31 APC | Thermo Fisher | 17-0311-82 | 1/100 |
| Rabbit monoclonal anti-WT1 | Abcam | Ab89901 | 1/200 |
| Mouse monoclonal anti-Tnnt2 | DSHB (Developmental Studies Hybridoma Bank) | CT3 | 1/20 |
| Chicken polyclonal anti-GFP | Abcam | Ab13970 | 1/200 |
| Mouse monoclonal anti-Smooth muscle actin | Sigma | A2547 | 1/100 |
| Mouse monoclonal anti-Myosin heavy chain, sarcomere | DSHB (Developmental Studies Hybridoma Bank) | MF20 | 1/25 |
| Mouse monoclonal anti-α-actinin | Sigma | A7811 | 1/200 |
| Rabbit polyclonal anti-Aldh1a2 (Raldh2) | Abcam | Ab75674 | 1/100 |
| Rabbit polyclonal anti-HCN4 | Alomone Labs | APC-052 | 1/100 |
| Mouse monoclonal anti-BrdU | DSHB (Developmental Studies Hybridoma Bank) | G3G4 | 1/100 |
| Lectin from *Bandeiraea simplicifolia* | Sigma | L5264 | 1/100 |

**Supplementary table 2:** Primers used in this study

| Gene | qPCR Primers |
| --- | --- |
| Acvr1c | Fw: CAGAATCCTAAAGTGGGAAC  Rv: CATGTCATAGTAAGGCAACTG |
| Calr4 | Fw: GGATTAAGAAAGGATGTGGAG  Rv: CTTTTCCCTCTTCTTCTTGG |
| Kcne1l | Fw: AGAAGATGATGAGAGGTCAG  Rv: TTAATTCGGTTGGATATGCG |
| Kcnab1 | Fw: ATCATTGAAGGACTGAAAGG  Rv: GAGTAGGCTTCCATGATCTC |

**Supplementary table 3: List of 137 genes differentially expressed in the RNASeq analysis at E13.5 (green: upregulated in mutant, red: downregulated in mutant).**

| **Gene_symbol** | **ENTREZID** | **baseMean** | **log2FoldChange** | **lfcSE** | **stat** | **pvalue** | **padj** |
| --- | --- | --- | --- | --- | --- | --- | --- |
| Cldn3 | 12739 | 333,6944556 | -3,172197944 | 0,330691426 | -9,592622298 | 8,58742E-22 | 1,77485E-17 |
| Kcne1l | 66240 | 555,066239 | 1,902146574 | 0,223417754 | 8,513855933 | 1,68243E-17 | 1,72127E-13 |
| Adcyap1r1 | 11517 | 539,0124188 | 2,623244393 | 0,309786708 | 8,467904926 | 2,49846E-17 | 1,72127E-13 |
| Tnxb | 81877 | 355,8576571 | 2,678054946 | 0,323835342 | 8,269804441 | 1,34179E-16 | 6,93302E-13 |
| Pou3f2 | 18992 | 48,08841672 | -4,467332851 | 0,596576171 | -7,488285773 | 6,9779E-14 | 2,88438E-10 |
| Rspo1 | 192199 | 353,5578463 | 1,782524052 | 0,240197738 | 7,421069261 | 1,16179E-13 | 4,00196E-10 |
| Shox2 | 20429 | 1548,149331 | 1,042102131 | 0,145956702 | 7,139803217 | 9,34646E-13 | 2,75961E-09 |
| Prrg4 | 228413 | 149,6834924 | -2,225966743 | 0,316214288 | -7,039424933 | 1,93035E-12 | 4,98706E-09 |
| Col8a1 | 12837 | 519,48807 | -1,668893992 | 0,24398004 | -6,840289019 | 7,90336E-12 | 1,81496E-08 |
| Lsp1 | 16985 | 3148,627909 | 0,961996557 | 0,14153042 | 6,797100968 | 1,06745E-11 | 2,20621E-08 |
| Trim72 | 434246 | 668,7952115 | -1,103747615 | 0,168038746 | -6,568411396 | 5,08549E-11 | 9,55517E-08 |
| Msr1 | 20288 | 233,927013 | -1,295535342 | 0,199476203 | -6,494686196 | 8,32067E-11 | 1,4331E-07 |
| Tcf21 | 21412 | 1986,193577 | 1,102747657 | 0,172373648 | 6,397426015 | 1,58018E-10 | 2,51224E-07 |
| Car4 | 12351 | 111,7580131 | 2,316314511 | 0,39671992 | 5,838664487 | 5,26209E-09 | 7,76835E-06 |
| Ppbp | 57349 | 223,2223985 | -2,025318389 | 0,354007302 | -5,721120383 | 1,05824E-08 | 1,45811E-05 |
| Timd4 | 276891 | 60,18020606 | -2,692220101 | 0,471753325 | -5,706838638 | 1,15094E-08 | 1,48672E-05 |
| Fndc1 | 68655 | 579,1349391 | -1,736243827 | 0,311371612 | -5,57611471 | 2,4595E-08 | 2,70199E-05 |
| Tmtc1 | 387314 | 1650,348077 | -0,778733784 | 0,139698366 | -5,574394376 | 2,48392E-08 | 2,70199E-05 |
| Ap3b2 | 11775 | 468,9094058 | 1,302799326 | 0,233332015 | 5,583457219 | 2,35784E-08 | 2,70199E-05 |
| Serpine1 | 18787 | 468,3614304 | -1,094403369 | 0,199146186 | -5,495477434 | 3,89655E-08 | 4,02669E-05 |
| Smpd3 | 58994 | 802,6683516 | -1,605733701 | 0,293164907 | -5,477237079 | 4,32018E-08 | 4,25188E-05 |
| Socs2 | 216233 | 2698,292375 | -0,741046986 | 0,136074045 | -5,445909899 | 5,15412E-08 | 4,84206E-05 |
| Slc15a3 | 65221 | 145,7302416 | -1,25709619 | 0,232757074 | -5,400893605 | 6,63097E-08 | 5,71037E-05 |
| Ntf5 | 78405 | 217,6335456 | 1,388207113 | 0,256925544 | 5,403149454 | 6,54809E-08 | 5,71037E-05 |
| Vsnl1 | 26950 | 1779,62677 | 0,996705596 | 0,187090221 | 5,327406162 | 9,96252E-08 | 8,23622E-05 |
| Kirrel2 | 243911 | 38,1502079 | 2,967594314 | 0,55841032 | 5,314361515 | 1,07032E-07 | 8,50822E-05 |
| Mpeg1 | 17476 | 751,1555949 | -0,864384872 | 0,164690602 | -5,24853794 | 1,53311E-07 | 0,000117357 |
| Plek | 56193 | 682,6400458 | -0,989417358 | 0,189699951 | -5,215696442 | 1,83128E-07 | 0,000135175 |
| Ccr5 | 12774 | 129,5702349 | -1,36787869 | 0,262857872 | -5,203871892 | 1,95179E-07 | 0,000139102 |
| Gjd2 | 14617 | 25,03359368 | -4,216861315 | 0,829982818 | -5,080660978 | 3,76124E-07 | 0,000259124 |
| Krt8 | 16691 | 1625,096164 | -1,133400104 | 0,223426569 | -5,072808074 | 3,91988E-07 | 0,000261342 |
| Kcna5 | 16493 | 945,9917557 | 0,71827938 | 0,142368514 | 5,045212309 | 4,53018E-07 | 0,000292593 |
| C1qa | 12259 | 1048,626121 | -0,985783559 | 0,196810628 | -5,008792303 | 5,47726E-07 | 0,000343043 |
| Apela | 100038489 | 447,838981 | 1,001407122 | 0,200362976 | 4,997964909 | 5,79385E-07 | 0,000352198 |
| Calb2 | 12308 | 61,87601983 | 1,984738523 | 0,399805612 | 4,964258794 | 6,89639E-07 | 0,000407242 |
| Ntng2 | 171171 | 43,01243421 | 2,238104014 | 0,4522261 | 4,949081922 | 7,45644E-07 | 0,000428082 |
| Acvr1c | 269275 | 297,072383 | 1,38413266 | 0,280753638 | 4,930061345 | 8,22038E-07 | 0,000459186 |
| Vsig4 | 278180 | 16,99213851 | -4,351513728 | 0,887241515 | -4,904542509 | 9,36453E-07 | 0,000468552 |
| Serpina9 | 71907 | 90,8325196 | -2,610953157 | 0,53181794 | -4,909486801 | 9,1315E-07 | 0,000468552 |
| Bst1 | 12182 | 57,7537933 | -1,951286064 | 0,398104406 | -4,901443027 | 9,51352E-07 | 0,000468552 |
| Rn7sk | 19817 | 70,79513272 | 1,689745487 | 0,344756177 | 4,9012769 | 9,52157E-07 | 0,000468552 |
| Trpa1 | 277328 | 20,28078594 | 3,475175301 | 0,706443 | 4,919257887 | 8,6873E-07 | 0,000468552 |
| Siglec1 | 20612 | 63,18052411 | -1,95656664 | 0,400336228 | -4,88730847 | 1,02224E-06 | 0,000488858 |
| Lgals3 | 16854 | 129,916073 | -1,503677003 | 0,307892236 | -4,883776946 | 1,04073E-06 | 0,000488858 |
| Csdc2 | 105859 | 279,2196419 | 0,923937247 | 0,190375864 | 4,8532268 | 1,21469E-06 | 0,000557892 |
| Vat1l | 270097 | 319,8142381 | -1,607768488 | 0,336734779 | -4,774583999 | 1,80079E-06 | 0,000809103 |
| Ctss | 13040 | 919,1007214 | -0,729976107 | 0,153858186 | -4,74447362 | 2,09049E-06 | 0,000919283 |
| 2610028E06Rik | 72395 | 14,06844003 | 7,257508934 | 1,534020062 | 4,731039126 | 2,23373E-06 | 0,000961809 |
| Tubb1 | 545486 | 166,1330863 | -1,320852832 | 0,282351525 | -4,678043916 | 2,89625E-06 | 0,001197192 |
| Ddc | 13195 | 4939,404758 | -0,825164238 | 0,176369195 | -4,678618848 | 2,88814E-06 | 0,001197192 |
| Dact2 | 240025 | 480,4026453 | 0,78848748 | 0,169123586 | 4,662197034 | 3,12851E-06 | 0,001267846 |
| Gja1 | 14609 | 27946,77454 | -0,669627437 | 0,144048934 | -4,648610865 | 3,34178E-06 | 0,001328229 |
| Col8a2 | 329941 | 804,3980644 | 1,088829723 | 0,234644253 | 4,640342608 | 3,47832E-06 | 0,001356414 |
| Zfyve28 | 231125 | 63,82921074 | -1,563130991 | 0,339277251 | -4,607237844 | 4,08053E-06 | 0,001561786 |
| Gpnmb | 93695 | 48,26520279 | -1,817853095 | 0,399329771 | -4,552260381 | 5,30726E-06 | 0,001994372 |
| Ptprz1 | 19283 | 149,7634208 | -1,568676112 | 0,347095542 | -4,519436068 | 6,20046E-06 | 0,002288411 |
| Cacng6 | 54378 | 1625,645381 | -0,950406648 | 0,211270183 | -4,49853659 | 6,84228E-06 | 0,002480987 |
| Ptprt | 19281 | 49,05221365 | 1,99775644 | 0,446513184 | 4,474126433 | 7,67243E-06 | 0,002734032 |
| Calca | 12310 | 273,7033913 | 1,215710156 | 0,272490502 | 4,461477176 | 8,13966E-06 | 0,002851365 |
| Kcnab1 | 16497 | 300,8453925 | -1,315814093 | 0,295242896 | -4,456717199 | 8,32243E-06 | 0,002866799 |
| Them7 | 74088 | 77,33922582 | -1,433495329 | 0,322503632 | -4,444896692 | 8,79341E-06 | 0,002979381 |
| Fcna | 14133 | 273,3897344 | -3,182612268 | 0,718606441 | -4,42886688 | 9,47295E-06 | 0,003157853 |
| Arg1 | 11846 | 110,3692744 | -2,371057994 | 0,5411932 | -4,381167381 | 1,18045E-05 | 0,003753472 |
| C1qb | 12260 | 1268,901746 | -0,777098255 | 0,177242321 | -4,384383202 | 1,16315E-05 | 0,003753472 |
| Samd9l | 209086 | 300,459202 | -0,768160154 | 0,175230293 | -4,383717785 | 1,16671E-05 | 0,003753472 |
| F2rl2 | 14064 | 49,13111822 | -2,148936085 | 0,494946843 | -4,341751273 | 1,41351E-05 | 0,004426444 |
| Kcnk2 | 16526 | 118,0476891 | -1,650554817 | 0,380597706 | -4,336743999 | 1,44609E-05 | 0,00446086 |
| Srgn | 19073 | 220,3866381 | -1,155909686 | 0,268669461 | -4,302348619 | 1,68997E-05 | 0,005136519 |
| Apoe | 11816 | 20092,07508 | -0,526163534 | 0,122831942 | -4,283605098 | 1,83889E-05 | 0,005508147 |
| Ccl6 | 20305 | 141,8263701 | -1,920722749 | 0,450228936 | -4,266102411 | 1,98917E-05 | 0,00579046 |
| Hapln4 | 330790 | 31,32965166 | 2,225068799 | 0,521425117 | 4,267283505 | 1,97868E-05 | 0,00579046 |
| Dpep1 | 13479 | 96,62447857 | 1,397570221 | 0,327974893 | 4,261210993 | 2,03322E-05 | 0,005836476 |
| Ryr3 | 20192 | 646,8052895 | 0,998783664 | 0,235286882 | 4,244961107 | 2,18631E-05 | 0,006189962 |
| Sla | 20491 | 190,9133546 | -0,881579624 | 0,208309587 | -4,232064578 | 2,31556E-05 | 0,006467294 |
| Wnt5b | 22419 | 669,3074201 | 0,662433904 | 0,15688022 | 4,22254575 | 2,41558E-05 | 0,006569116 |
| Ngfr | 18053 | 411,2647208 | 1,243004678 | 0,294346648 | 4,222927919 | 2,41149E-05 | 0,006569116 |
| Cybb | 13058 | 224,8409291 | -1,039056994 | 0,246671573 | -4,212309427 | 2,52773E-05 | 0,006711934 |
| Ccdc80 | 67896 | 7103,003138 | -0,60704504 | 0,144226963 | -4,2089567 | 2,56553E-05 | 0,006711934 |
| Mme | 17380 | 666,6054462 | 0,825605457 | 0,196099843 | 4,21012809 | 2,55226E-05 | 0,006711934 |
| Ttc22 | 230576 | 52,6602009 | 1,706422908 | 0,406217438 | 4,200762325 | 2,66018E-05 | 0,006872568 |
| Il17re | 57890 | 74,59635187 | -1,993835986 | 0,475962807 | -4,189058384 | 2,80114E-05 | 0,007147411 |
| Mirt1 | 381232 | 53,49304721 | -1,711975128 | 0,41671437 | -4,108269956 | 3,98634E-05 | 0,009693695 |
| Sema3e | 20349 | 180,1264831 | -1,440285101 | 0,350159829 | -4,11322197 | 3,90175E-05 | 0,009693695 |
| Alox12 | 11684 | 178,5730024 | -1,066388143 | 0,25957229 | -4,108251085 | 3,98667E-05 | 0,009693695 |
| Lamc3 | 23928 | 84,24827857 | 2,01661179 | 0,490698025 | 4,109679858 | 3,96208E-05 | 0,009693695 |
| Bcar3 | 29815 | 494,3461784 | -1,012324975 | 0,246590169 | -4,105293329 | 4,03802E-05 | 0,009704399 |
| Vegfd | 14205 | 529,7021072 | -1,031032994 | 0,251403285 | -4,101111865 | 4,1117E-05 | 0,00976788 |
| Tm4sf5 | 75604 | 152,2016774 | 1,234679176 | 0,301810707 | 4,090905815 | 4,29692E-05 | 0,010091894 |
| Nr2f1 | 13865 | 1597,205742 | 0,961409566 | 0,235319009 | 4,085558461 | 4,39709E-05 | 0,010211136 |
| Cish | 12700 | 788,4398417 | -0,578804322 | 0,142178347 | -4,070973773 | 4,6817E-05 | 0,010633123 |
| Sox3 | 20675 | 512,0258033 | 1,284130426 | 0,315344317 | 4,07215338 | 4,65805E-05 | 0,010633123 |
| Thy1 | 21838 | 241,1155547 | -1,081378413 | 0,265851369 | -4,067605208 | 4,74988E-05 | 0,010670701 |
| Tmem255a | 245386 | 617,996164 | 0,92108655 | 0,226731753 | 4,062450617 | 4,85602E-05 | 0,010791857 |
| Clu | 12759 | 9877,318903 | -0,608814003 | 0,150822835 | -4,036616891 | 5,42275E-05 | 0,01192313 |
| Krt17 | 16667 | 184,110224 | -1,189271163 | 0,295494085 | -4,024686869 | 5,70512E-05 | 0,012411934 |
| Tnxa | 100043024 | 11,841155 | 4,060248282 | 1,017619519 | 3,989947328 | 6,6088E-05 | 0,014228189 |
| Igfbpl1 | 75426 | 2872,562449 | 0,675531294 | 0,170384316 | 3,964750462 | 7,34728E-05 | 0,015655001 |
| Zic1 | 22771 | 38,96787512 | -1,85667276 | 0,469863434 | -3,951515749 | 7,76578E-05 | 0,01621243 |
| Tmem196 | 217951 | 136,6600229 | -1,153052037 | 0,291692224 | -3,952974888 | 7,71855E-05 | 0,01621243 |
| Cpne5 | 240058 | 1419,229975 | 0,635705775 | 0,160993071 | 3,948653014 | 7,85922E-05 | 0,01624343 |
| Clec7a | 56644 | 231,486934 | -1,378619317 | 0,349448104 | -3,945133202 | 7,97556E-05 | 0,016320687 |
| Arhgef5 | 54324 | 1393,058715 | -0,540828391 | 0,137251798 | -3,940410245 | 8,13424E-05 | 0,016482199 |
| Rasef | 242505 | 91,09806615 | 1,11729588 | 0,284650427 | 3,925150897 | 8,66754E-05 | 0,0173923 |
| Hydin | 244653 | 64,63282026 | 2,097715294 | 0,535503281 | 3,917278132 | 8,95544E-05 | 0,017627718 |
| Nphs1 | 54631 | 27,98249079 | 2,451147113 | 0,625604289 | 3,918047168 | 8,92692E-05 | 0,017627718 |
| Prph | 19132 | 79,72448894 | -1,449198529 | 0,370476496 | -3,911715172 | 9,1643E-05 | 0,017868647 |
| Ptpn3 | 545622 | 346,4629454 | -0,791569014 | 0,203594913 | -3,88796067 | 0,00010109 | 0,019526431 |
| Fst | 14313 | 435,2715767 | 0,857677543 | 0,221707428 | 3,868510638 | 0,000109502 | 0,020955469 |
| Lox | 16948 | 3854,404318 | -0,689905822 | 0,178663546 | -3,861480636 | 0,000112702 | 0,021369943 |
| Wnt2 | 22413 | 473,5013031 | 0,759643089 | 0,1974821 | 3,846642752 | 0,000119747 | 0,022499443 |
| D630003M21Rik | 228846 | 136,433666 | 1,220362267 | 0,318414194 | 3,832625206 | 0,000126783 | 0,023606773 |
| Gjb5 | 14622 | 32,94171287 | -2,111283762 | 0,551315369 | -3,8295391 | 0,000128383 | 0,023691338 |
| Parp3 | 235587 | 129,963307 | -0,931620564 | 0,243863561 | -3,820253263 | 0,000133315 | 0,024383615 |
| Serpinb1a | 66222 | 270,3463007 | -0,801430262 | 0,21049072 | -3,807437496 | 0,000140414 | 0,02545685 |
| Gm20744 | 434205 | 30,21143446 | -2,285364242 | 0,601407193 | -3,800028115 | 0,00014468 | 0,025777927 |
| Ddx25 | 30959 | 284,0208651 | 0,694592442 | 0,182740068 | 3,800986009 | 0,000144121 | 0,025777927 |
| Slc38a5 | 209837 | 477,8198112 | 0,723880545 | 0,191945555 | 3,771280588 | 0,000162412 | 0,028689997 |
| Calr4 | 108802 | 17,12688349 | 2,909472925 | 0,774337031 | 3,757372836 | 0,000171707 | 0,030074843 |
| Casq2 | 12373 | 12637,98159 | -0,881470684 | 0,235149844 | -3,74854888 | 0,000177861 | 0,030890962 |
| Gp6 | 243816 | 31,13422527 | -1,875125143 | 0,501698415 | -3,73755445 | 0,000185819 | 0,031787785 |
| Stat3 | 20848 | 4720,405488 | -0,629663794 | 0,168573965 | -3,735237498 | 0,000187538 | 0,031787785 |
| Pitpnm3 | 327958 | 65,11836895 | 1,338005643 | 0,358224563 | 3,735103011 | 0,000187638 | 0,031787785 |
| Ppp1r14c | 76142 | 15188,43179 | -0,435159194 | 0,116941869 | -3,721158178 | 0,000198311 | 0,033322725 |
| Cd226 | 225825 | 37,00737018 | -1,971561936 | 0,533061694 | -3,698562399 | 0,000216824 | 0,036139673 |
| Muc16 | 73732 | 86,22201257 | 1,838840286 | 0,499676331 | 3,680062815 | 0,000233177 | 0,038554336 |
| Aldh1a1 | 11668 | 506,9047801 | -1,029397237 | 0,27992024 | -3,67746625 | 0,000235562 | 0,038639684 |
| Sowahd | 245381 | 25,05806248 | 2,617884536 | 0,71238247 | 3,674830087 | 0,000238008 | 0,03873342 |
| Lcp2 | 16822 | 151,5308018 | -0,89628668 | 0,244647505 | -3,66358398 | 0,000248711 | 0,040158993 |
| Cfi | 12630 | 143,1617851 | 1,431390127 | 0,3922622 | 3,64906465 | 0,000263197 | 0,042168627 |
| 6430584L05Rik | 330324 | 5,443152962 | 5,880426965 | 1,620335176 | 3,629142323 | 0,000284364 | 0,045209571 |
| Col1a1 | 12842 | 44772,54682 | -0,957107336 | 0,263910886 | -3,626630762 | 0,000287143 | 0,045302905 |
| Cacng7 | 81904 | 572,7378262 | 0,855674527 | 0,236366217 | 3,620121933 | 0,000294464 | 0,046105958 |
| Gatm | 67092 | 1867,924944 | -0,514265572 | 0,142353465 | -3,612596091 | 0,000303147 | 0,047108543 |
| Prr16 | 71373 | 52,9843973 | 1,288405528 | 0,357886063 | 3,600043868 | 0,000318163 | 0,049073158 |
| Mybphl | 68753 | 1389,818439 | 0,755432793 | 0,209974313 | 3,597739094 | 0,000320995 | 0,049143203 |
| Cyp2s1 | 74134 | 316,3499046 | -1,078008171 | 0,300166013 | -3,591373185 | 0,00032894 | 0,04998924 |
